# Dishevelled 2 regulates cancer cell proliferation and T cell mediated immunity in HER2-positive breast cancer

**DOI:** 10.1186/s12885-023-10647-2

**Published:** 2023-02-21

**Authors:** Fahmida Rasha, Geetha Priya Boligala, Mingxiao V. Yang, Dalia Martinez-Marin, Isabel Castro-Piedras, Kathryn Furr, Annie Snitman, Sonia Y. Khan, Luis Brandi, Maribel Castro, Hafiz Khan, Nusrat Jahan, Sharilyn Almodovar, Michael W. Melkus, Kevin Pruitt, Rakhshanda Layeequr Rahman

**Affiliations:** 1 Department of Immunology and Molecular Microbiology, Texas Tech University Health Sciences Center, 3601 4th Street, Lubbock, TX 79430 USA; 2 Depart of Cell Biology and Biochemistry, Texas Tech University Health Sciences Center, Lubbock, TX USA; 3 Department of Surgery, Texas Tech University Health Sciences Center, School of Medicine, 3601 4th Street, Lubbock, TX 79430 USA; 4 Breast Center of Excellence, Texas Tech University Health Sciences Center, Lubbock, TX USA; 5 Department of Pathology, Texas Tech University Health Sciences Center, Lubbock, TX USA; 6 Department of Public Health, Julia Jones Matthews, Texas Tech University Health Sciences Center, Lubbock, TX USA; 7 Department of Medicine, Texas Tech University Health Sciences Center, Lubbock, TX USA

**Keywords:** Breast cancer, Dishevelled, HER2, Tumor infiltrating lymphocytes, Wnt signaling

## Abstract

**Background:**

Dishevelled paralogs (DVL1, 2, 3) are key mediators of Wnt pathway playing a role in constitutive oncogenic signaling influencing the tumor microenvironment. While previous studies showed correlation of β-catenin with T cell gene expression, little is known about the role of DVL2 in modulating tumor immunity. This study aimed to uncover the novel interaction between DVL2 and HER2-positive (HER2+) breast cancer (BC) in regulating tumor immunity and disease progression.

**Methods:**

DVL2 loss of function studies were performed with or without a clinically approved HER2 inhibitor, Neratinib in two different HER2+ BC cell lines. We analyzed RNA (RT-qPCR) and protein (western blot) expression of classic Wnt markers and performed cell proliferation and cell cycle analyses by live cell imaging and flow cytometry, respectively. A pilot study in 24 HER2+ BC patients was performed to dissect the role of DVL2 in tumor immunity. Retrospective chart review on patient records and banked tissue histology were performed. Data were analyzed in SPSS (version 25) and GraphPad Prism (version 7) at a significance *p* < 0.05.

**Results:**

DVL2 regulates the transcription of immune modulatory genes involved in antigen presentation and T cell maintenance. DVL2 loss of function down regulated mRNA expression of Wnt target genes involved in cell proliferation, migration, invasion in HER2+ BC cell lines (±Neratinib). Similarly, live cell proliferation and cell cycle analyses reveal that DVL2 knockdown (±Neratinib) resulted in reduced proliferation, higher growth arrest (G1), limited mitosis (G2/M) compared to non-targeted control in one of the two cell lines used. Analyses on patient tissues who received neoadjuvant chemotherapy (*n* = 14) further demonstrate that higher DVL2 expression at baseline biopsy pose a significant negative correlation with % CD8α levels (*r* = − 0.67, *p* < 0.05) while have a positive correlation with NLR (*r* = 0.58, *p* < 0.05), where high NLR denotes worse cancer prognosis. These results from our pilot study reveal interesting roles of DVL2 proteins in regulating tumor immune microenvironment and clinical predictors of survival in HER2+ BC.

**Conclusion:**

Our study demonstrates potential immune regulatory role of DVL2 proteins in HER2+ BC. More in-depth mechanistic studies of DVL paralogs and their influence on anti-tumor immunity may provide insight into DVLs as potential therapeutic targets benefiting BC patients.

**Supplementary Information:**

The online version contains supplementary material available at 10.1186/s12885-023-10647-2.

## Introduction

Despite the improvement in 5-year survival rate in the past 3 decades, breast cancer remains the leading cause of cancer death among women [1]. Cancer cells escape the host immune response for the development and progression of cancer [2]. In traditional high-risk profiles of breast cancer such as triple-negative and HER2 positive cancers, higher percentage of tumor infiltrating lymphocytes (TILs) are associated with improved clinical outcome [3–5]. Intuitively, inhibition of TILs and their activity in the tumor microenvironment may impair antitumor immune response and limit treatment responsiveness in breast cancer.

Wnt pathway is pivotal in the development and function of hematopoietic system [6]. Dishevelled (DVL) proteins act as a branch point for integrating and transmitting signals in both canonical (β-catenin dependent) and non-canonical (β-catenin independent) Wnt pathways and help maintain constitutive oncogenic signaling. β-catenin, a key component in Wnt signaling, serves as a convergence point between the canonical Wnt and human epidermal growth factor receptor 2 (HER2) signaling pathways [7, 8]. In peripheral immunity, canonical Wnt/β-catenin signaling is critical for the polarization, differentiation and survival of mature T lymphocytes [9]. Activation of Wnt/β-catenin pathway could impair the naive-to-effector T cell differentiation in human T lymphocytes [10, 11]. In addition to affecting immune response, aberrant Wnt signaling contributes to the initiation and progression of various human cancers including breast cancer. From immunosuppression of the tumor microenvironment (TME) and reduction in TIL activity to modulating several cancer hallmarks such as unrestrained growth, increased neoplastic cell survival, invasion, and metastasis, the overactivity of the Wnt pathway is critical for tumor progression [7]. Though DVL proteins are significant drivers of oncogenic Wnt signaling, little is known about their mechanistic interactions with the HER2 signaling pathway and their ultimate role in modulating tumor immunity and disease progression in HER2-positive (HER2+) breast cancers.

TILs¸ mononuclear immune cells located in tumor tissues that can recognize and kill cancer cells [12], are crucial elements of the TME and reflect host anti-tumor immune response [4]. Key markers of tumor immune response include TIL score, levels of cytotoxic (CD8α) T-cells, neutrophil or platelet to lymphocyte ratio (NLR/PLR). It has been demonstrated that TILs and other measurable factors may prove useful as predictive and prognostic biomarkers for several breast cancer subtypes [13, 14]. Immunologic invasion involving both innate and adaptive immune responses is key to blocking tumor progression, and understanding the molecular mechanism involved in evasion of immune activation. Moreover, each 10% increment in stromal TILs was associated with an 18% increase in overall survival and a 17% decrease in risk of recurrence in HER2+ and Triple negative breast cancer (TNBC) patients, respectively [5, 15]. In comparison, ER-positive and HER2-negative tumors have less TIL infiltration and do not demonstrate similar survival benefits as HER2+ and TNBCs [16], since higher ER expression can potentially promote anti-inflammatory Th2 type immune response and in turn, reduce cytotoxicity in the tumors [17]. Moreover, TME rich in regulatory T-cells or tumor-associated macrophages (TAMs) reprogrammed by the tumor itself inhibit lymphocyte functions through macrophage polarization and the release of inhibitory cytokines such as interleukin (IL)-10, prostaglandins, and reactive oxygen species (ROS) [18]. This contributes to a pro-inflammatory milieu in the tumor stroma favoring tumor survival and evolves into a phenotype allowing cytotoxic immune escape [19]. Additionally, activation of distinct tumor intrinsic pathways such as, the Wnt/β-catenin pathway is associated with an immune-cold tumor TME and low TIL infiltration in melanoma and colorectal cancer [20–22] while high levels of stromal TIL infiltration were found to be correlated with β-catenin overexpression in breast cancer, raising the question of involvement of Wnt/β-catenin/DVL signaling cascades in tumor immune-surveillance and anti-tumor immune response.

Therefore, we hypothesize that (i) DVL2 depletion will reduce the expression of classic oncogenic Wnt markers involved in cancer cell proliferation and invasion in HER2-overexpressed in vitro models of breast cancer, and (ii) clinically, high DVL2 protein expression will modulate markers of TIL infiltrations and cytotoxicity such as TIL score, CD8α, and NLR/PLR, eventually resulting in poor survival outcomes in HER2+ breast cancer.

## Methods

### Human subjects and data collection

An IRB approved (TTUHSC IRB# L21-175) retrospective study was conducted to determine correlations between DVL2 protein expression with patient clinicopathological parameters and clinical survival predictors. Patient data were collected for 24 HER2+ breast cancer patients using the electronic health records at the University Medical Center (UMC) Cancer Center in Lubbock, TX, USA. Patient tumor biopsy samples were sectioned and analyzed for DVL2 and CD8α expression by immunofluorescence. Hematoxylin and eosin (H&E)-stained biopsy sections were analyzed by two expert pathologists for prognostic factors such as TIL-scores, NLR and PLR due to their association with pathological complete response (pCR) – long term survival rates. DVL2 expression was then evaluated by spearman’s rank correlation to test strength and direction of correlation between to each of the following parameters: age, BMI, ER status, PR status, percentage Ki67, CD8a, TIL-score, tumor NLR and PLR. *p* < 0.05 was considered significant.

### Residual cancer burden index (RCBI) calculation

We used the published method to evaluate the pathology materials and the associated web site (http://www3.mdanderson.org/app/medcalc/index.cfm?pagename=jsconvert3) to calculate RCB [23]. Briefly, the gross description (including radiographs, diagrams, or photographs) and all corresponding slides underwent a retrospective review by two expert pathologists to determine the RCB index and class after entering appropriate data in the interactive web-based calculator.

All the data concerning patients’ clinical and pathological features, along with the type of treatment administered and related outcome, were retrospectively collected and entered into an anonymized dedicated database.

### UALCAN analysis of DVL2 expression in breast cancer

UALCAN (http://ualcan.path.uab.edu) is a comprehensive and user-friendly-interactive web resource for analyzing cancer OMICS data (TCGA, MET500, CPTAC, and CBTTC) [24]. We used UALCAN to plot graphs depicting DVL2 protein expression based on breast cancer sample types (normal vs. primary tumor) and major subclasses (normal, luminal, HER2+ and TNBC).

### Immunofluorescence staining using FFPE primary human tissues

Standard H&E staining of fixed paraffin-embedded tissue (FFPE) was performed for histopathological examination and TIL scoring. Images were taken using a Leica Model DM 2000 LED microscope (Buffalo Grove, IL) equipped with an MC 170 HD camera and Leica Application Suite version 3.4 software and TIL scoring was performed independently by two trained professionals and mean value was taken for further analysis. FFPE BC tissue sections were stained to identify the expression of DVL2 (LSBio#375617; 1:200) and CD8α (Abcam#; 1:100) by manufacturer’s protocol. The slides were blocked at room temperature with 1% phosphate buffer saline (PBS) (Thermo Fisher Scientific, Waltham, MA USA), 0.1% Saponin (Millipore Sigma, Burlington MA, USA), and 5% bovine serum albumin (BSA) (Millipore Sigma, Burlington MA, USA) for 1 h and then incubated overnight at 4 °C with DVL2 and CD8α in previously mentioned blocking buffer. Next day the slides were incubated with Alexa fluorophore conjugated secondary Cy3TM donkey anti-rabbit IgG (Thermo Fisher Scientific) at 1:300 dilution for 2 h at room temperature and mounted with DAPI (Life Technologies, Carlsbad, CA, USA) overnight in the dark to allow hardening. The slides were sealed with nail polish and stored at 4 °C for longer time-period. The images were taken at 60X and 40X magnification using a laser scanning confocal microscope Nikon T-1E. The images were then analyzed using AdipoGauge software for Windows (Version 2.0) [25]. Antibody fluorescence was normalized using TRITC/DAPI staining for each sample and Log2 fold change values were calculated to examine further statistical association and/or correlation.

### Correlations between DVL2 expression and survival in HER2+ breast cancer

We utilized the Kaplan Meier Plotter (https://kmplot.com/analysis), a web-based meta-analysis tool to assess correlation between gene expression and survival from 21 tumor types (GEO, EGA, and TCGA) for discovery and validation of survival biomarkers [26]. Here, we compare the expression of DVL2 and survival status in HER2+ breast cancer (*n* = 282). Overall survival (OS) and distant metastases-free survival (DMFS) were evaluated at a hazard ratio (HR) of 95% confidence interval and *p*-value less than 0.05 were considered statistically significant.

### In silico analysis

Removal of sequencing adapters, low-quality bases and reads, and unpaired reads from previously described DVL3 ChIP samples [27] was first performed by using fastqc. Bowtie2 was used for the alignment of preprocessed reads to the hg38/GRCh38 reference genome. Mapping files in sam format were converted to files in bam format by using Samtools version1.5 followed by bamCoverage application of deepTools version2.0 for calculating reads intensity across the genome. DNASTAR’s Laser Gene software and Integrative Genomics Viewer (IGV) was used to visualize sequencing read intensity profiles at specific regions.

### The Cancer Genome Atlas (TCGA) data analysis

Genomic data from Breast Invasive Carcinoma (BRCA, TCGA, PanCancer Atlas) study was analyzed using the cBio Cancer Genomics Portal (http://cbioportal.org). The study consisted of gene expression analysis from 1084 breast cancer patients [28]. Correlation analysis was performed between selective immune modulatory genes and DVL2 in different breast cancer subtypes (data not shown). A selective immune-modulatory gene set were chosen based on DVL-3 ChIP-Seq hits in TNBC cell line using methodologies previously published by our lab [27, 29].

### Cell culture experiments

Two HER2+ human breast cancer cell lines used in this manuscript (SKBR3, and BT474) were purchased from ATCC. The cells were authenticated utilizing short tandem repeat (STR) DNA profiling and used in a low passage (< 20) within 6 months after receipt or resuscitation. SKBR3 (ER/PR-negative, HER2-positive) cells were grown in McCoy’s 5A medium (Gibco), and BT474 (ER/PR/HER2-positive) cells were grown in Ham’s F-12 DMEM nutrient mix medium (Gibco), supplemented with 10% fetal bovine serum and 1% penicillin/streptomycin (Invitrogen) [30].

### DVL2 stable knockdown

HER2+ breast cancer cells were transduced with pLKO.1-puro based shRNA MISSION lentiviral particles for DVL2, shDVL2 (TRCN000033340, Sigma), and non-target shRNA control, NTC (SHC002V, Sigma). After 24 h of plating, cells were transduced with hexadimethrine bromide (Sigma, H9268) at a final concentration of 8 μg/ml, followed by addition of appropriate amounts of viral particles to the media. After 24 h, the media was replaced and 1 μg/ml of puromycin (Gibco, A11138-03) was added for selection. Puromycin-containing media was replaced every 3–4 days until total selection was achieved [30]. Supplementary Fig. S1A and B respectively demonstrate the selection process of SKBR3 and BT474 clonal cell lines.

### Neratinib treatment

Neratinib (HKI-272), a clinically approved highly selective HER2 and epidermal growth factor receptor (EGFR) inhibitor, was purchased commercially (Selleckchem). Time dependent dose response studies were performed in both cancer cell lines (data not shown) to select the final dose of 1 nM. NTC and shDVL2 stable cell lines were treated with and without 1 nM for 24 h to investigate the combined effect of DVL2 loss of function and HER2 inhibition in two different HER2+ breast cancer cell lines. RNA and protein samples were collected, and cell cycle analyses were performed.

### Subcellular fractionation

SKBR3 and BT474 parental cells were used for nuclear and cytoplasmic fractionation of DVL2 protein localization. Nuclear and cytosolic extracts were prepared using the NE-PER kit (Thermo Scientific).

### Fluorescence microscopy

We followed previously published protocol by our laboratory for fluorescence microscopy [31]. The DVL2 primary antibody was used at a 1:200 dilution (LSBio#375617); while the secondary antibodies: Alexa flour 568 #A11036 and phalloidin 488 #A12379 were used at 1:300 dilution (Thermo Scientific). Images were taken by Nikon confocal TIE inverted microscope at a 60X magnification.

### Immunoblots

Antibodies used for immunoblots are listed in Supplementary Table 10. Membranes were incubated with blocking buffer (5% milk/TBST) with primary antibody overnight at 4 °C followed by probing the membranes with HRP-conjugated secondary antibodies for 1 h at room temperature. Lastly, the signals were visualized using ECL reagent (Thermo Scientific) and imaged in Azure C300 gel imaging system (Azure Biosystems). Immunoblot data were quantified using NIH Image J software for Windows [30].

### mRNA expression analysis, end-point PCR, and quantitative real-time qRT-PCR

Total RNA was isolated from cells using a Bio-Rad Aurum Total RNA extraction kit and 2 μg of RNA was reverse-transcribed using SuperScript III Reverse Transcriptase (ThermoFisher) to synthesize first-strand of complementary DNA (cDNA). Intron-spanning primers were designed (Supplementary Table 9) using NIH primer design website and gene expression measured by either endpoint-PCR using JumpStart RedTaq (Sigma) or real-time qRT-PCR SYBR Green MasterMix (BioRad). The Gel DOC EZ imager (Bio-Rad) and the Applied Biosystems Veriti 96-well thermal cycler (Applied Biosystems) and were used for analyses respectively.

### Chromatin immunoprecipitation (ChIP)

ChIP was done according to previously published laboratory protocol [31]. Cells were grown to confluence at a final count of approximately 5 million cells per plate. Cells were cross-linked with 1% (w/v) formaldehyde for 8 min and quenched in 0.125 M of glycine for 5 min at room temperature. The media was removed, and cells were washed twice with sterile ice-cold 1X PBS. Cells were scraped in 1X PBS plus protease inhibitor cocktail and centrifuged to obtain cell pellet. Pelleted cells were resuspended in SDS Lysis buffer (50 mM Tris-HCl pH 8.0, 10 mM 0.5 M EDTA, and 1% SDS) with protease inhibitor cocktail and were then sonicated in a Diagenode Bioruptor sonicator for 25 and 35 cycles (30 sec pulses and 30 sec rest) for SKBR3 and BT474 cells, respectively. The soluble chromatin fraction was quantitated and 100 μg of chromatin was immunoprecipitated with anti-DVL2 antibody (LSBio#375617), or Rabbit IgG (I5006; Sigma) for 2 h at 4 °C. Dynabeads Protein A (Invitrogen, 10002D) were added to the chromatin-antibody mixture and incubated with rotation for 2 h at 4 °C. ChIPs were washed five times with low salt wash buffer (0.1% SDS, 1% Triton X-100, 2 mM EDTA, 20 mM Tris HCl pH 8.1, and 150 mM NaCl), three times with high salt wash buffer (0.1% SDS, 1% Triton X-100, 2 mM EDTA, 20 mM Tris HCl pH 8.1, and 500 mM NaCl), and once with TE (1 mM EDTA and 10 mM Tris HCl pH 8). Immunoprecipitated chromatin samples were reverse crosslinked overnight at 65 °C, followed by RNAse A (Promega) at 37 °C for 2 h, and proteinase K incubation (Promega) at 55 °C for 2 h. DNA was eluted using Qiaquick PCR purification kit (Qiagen) and amplified by end-point PCR using gene-specific primers as listed in Supplementary Table 9.

### Cell proliferation studies

HER2+ breast cancer cells stably expressing DVL2 knockdown, or NTC were maintained in their corresponding cell growth media. Cells were seeded at a density of 60,000-80,000 cells per well in 96 well plates and upon confluency, were treated with or without 1 nM neratinib. Cells were monitored in real-time by live-cell imaging at 37 °C, 5% CO2 and cell confluence was quantified using the IncuCyte ZOOM software (Sartorius). Images were collected every 3 h for 144 to 280 h (~ 6 to 12 days).

### Cell cycle analysis

HER2+ breast cancer cells stably expressing DVL2 knockdown, or NTC were seeded at a density of 5 million cells per 150mm^2^ plate and after 24 h were treated with or without 1 nM neratinib. For each cell type, 1 million cells per mL were stained according to the manufacturer’s protocol with Vybrant DyeCycle™ Violet Stain (Invitrogen) at a concentration of 5 μM, and data was collected on a BDFortessa. Collected data was then analyzed using FlowJo v7.6.5 (Becton Dickinson) [30].

### Neutrophil to lymphocyte ratio (NLR) and platelet to lymphocyte ratio (PLR) calculation

NLR and PLR was performed as previously described [32], all blood cell assessments were centrally performed in our institutional laboratory (University Medical Center) according to the standardized operative procedures. Peripheral complete blood count was performed at baseline, i.e. just before starting neoadjuvant chemotherapy (NAC). NLR was provided by the ratio between the absolute count of neutrophils and the absolute count of lymphocytes. PLR was calculated by dividing the absolute number of platelets by the absolute number of lymphocytes.

### Statistics

Statistical analysis was performed between two-four biological replicates using Welch’s independent t-test and differences were observed as Mean ± SEM. One-way Anova was performed to detect differences between more than two groups followed by Tukey’s post-hoc comparison. All results were considered significant at *p* < 0.05 and trend of significance reported at *p* ≤ 0.1. Descriptions for in vitro analyses can also be found in respective figure legends. For patient data analyses, unpaired or independent t-test were performed to record association between DVL2 and other clinical variables. Association between before and after NAC specimen was identified using Paired t-test and Spearman’s rank correlation test was performed to record correlation between DVL2 and other clinical variables. All statistical analyses were performed using SPSS Software version 25 (Armonk, NY, www.ibm.com) and results were considered significant at *p* < 0.05 and trend of significance reported at *p* ≤ 0.1. Graphs were generated using GraphPad Prism version 9.0 for Windows (La Jolla, CA, www.graphpad.com) and Morpheus software (https://software.broadinstitute.org/morpheus).

## Results

### DVL2 is highly expressed in HER2+ breast cancer and promotes poor survival outcomes

**Fig. 1:**
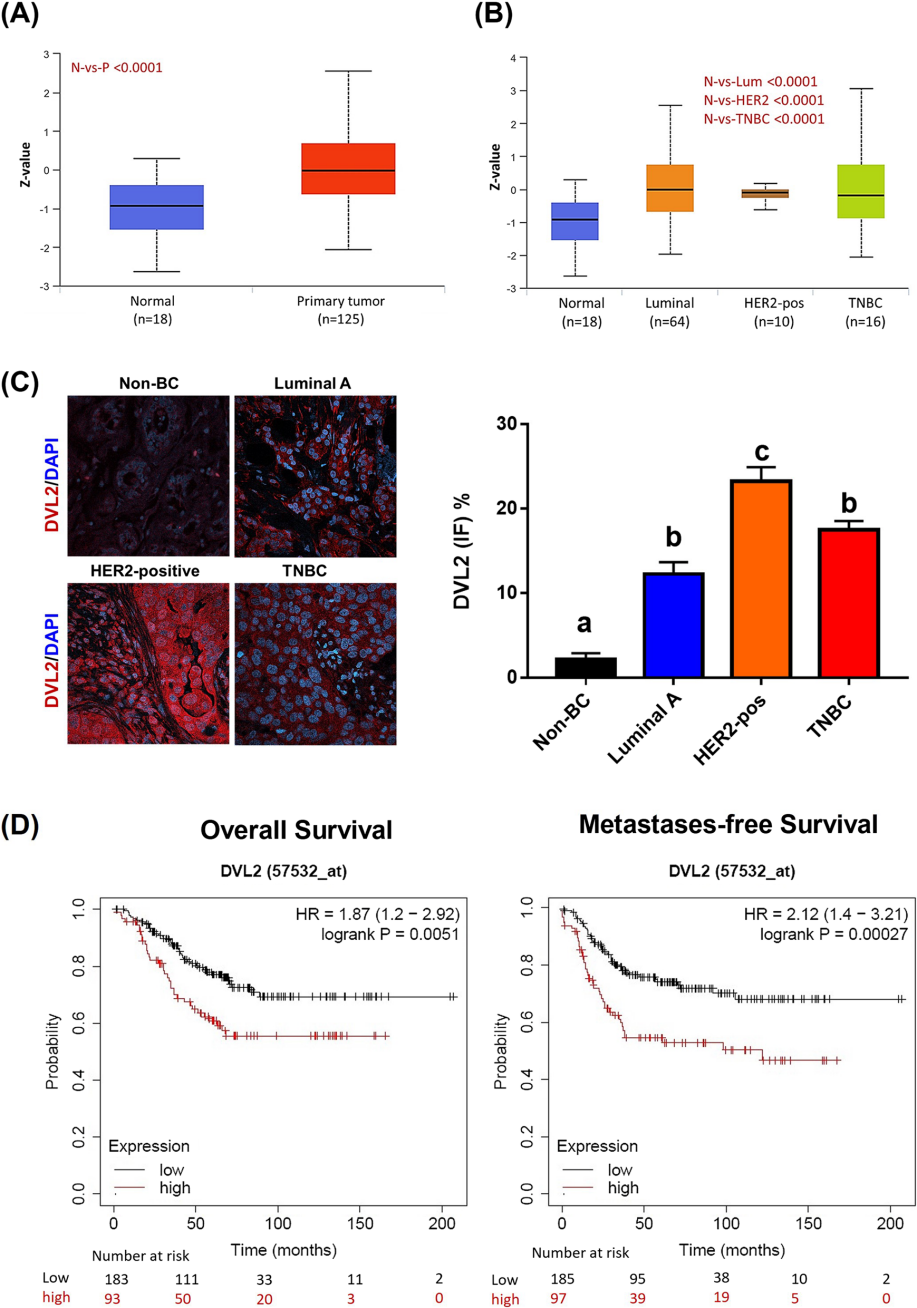
Higher Dishevelled (DVL) 2 expressions may predict lower survival in HER2-positive breast cancer patients. **A** UALCAN analysis for the comparison between DVL2 protein expressions in breast cancer using CPTAC samples in benign versus primary breast tumors. *P*-value significant codes: 0 ≤ *** < 0.001 ≤ ** < 0.01 ≤ * < 0.05 ≤ # < 0.1. **B** UALCAN analysis presenting respective DVL2 proteins expression in different subtypes of breast cancer (luminal, HER2-positive, and triple negative) using CPTAC samples. Z-values represent standard deviations from the median across samples for breast cancer sample/subclass types. Log2 Spectral count ratio values from CPTAC were first normalized within each sample profile, then normalized across samples. *P*-value significant codes: 0 ≤ *** < 0.001 ≤ ** < 0.01 ≤ * < 0.05 ≤ # < 0.1. **C** Immunofluorescent staining was performed to compare DVL2 protein expression in non-breast cancer (non-BC) versus different breast cancer subtypes (Luminal, HER2-positive, Triple negative) showing a merge of nuclear staining with DAPI (blue) and DVL2 (red) proteins. Images were taken from a small cohort of breast cancer patients (*N* = at least 5) in triplicates by a laser scanning confocal microscope Nikon T-1E with a 60x objective and NIS software and analyzed via AdipoGauge software (Windows version 2.0) [25] (*N* = 3, One-way Anova, different letters denote significance where *p* < 0.05). **D** Kaplan-Meier database was used to identify overall survival and distant metastasis-free survival comparing the high and low expressions of DVL2 (57532_at) in HER2-positive breast cancer (*n* = 276). CPTAC: Clinical Proteomic Tumor Analysis Consortium; N: normal; P: primary tumor; Lum: luminal; HER2: HER2-positive; TNBC: triple negative breast cancer

Dishevelled proteins are major regulators of oncogenic Wnt signaling and related tumor progression [33]. However, there is a lack of literature delineating mechanistic association of DVL2 in HER2+ breast cancer. Hence, we aim to understand the differential expression of DVL2 protein in different molecular subtypes of breast cancer. Using CPTAC breast cancer data cohort from UALCAN analysis platform, we confirmed that the expression of DVL2 in primary breast tumor samples (*n* = 125) was significantly higher than that in benign breast tissues (*n* = 18) (*p* < 0.0001) (Fig. 1A). Next, we found significantly higher DVL2 protein expression in the same cohort in different molecular subtypes of breast cancer: Luminal (*n* = 64), HER2+ (*n* = 10), TNBC (*n* = 16) compared to normal breast tissue (*n* = 18) (*p* < 0.0001). However, no significant changes of DVL2 protein expression were found across different subtypes of breast cancer in a separate subgroup analysis between Luminal, HER2+ and TNBC (Fig. 1B). Additionally, we performed immunofluorescence staining on FFPE tumor sections from patients with known molecular subtypes. Interestingly, DVL2 protein levels were significantly higher in all subtypes of breast cancer compared to benignbreast tissues and the highest expression levels were found in HER2+ tumors compared to other subtypes of breast cancer, Luminal and TNBC (Fig. 1C). Given this promising proteomic data, we next investigated the association between DVL2 expression and patient survival. Using Kaplan Meier survival curves, we compared whether high and low expression levels of DVL2 have any association with overall survival (OS) and distant metastases-free survival (DMFS) in HER2+ breast cancer patients. Remarkably, we observed that high DVL2 expression was significantly associated with both poor OS and DMFS with a hazard ratio of 1.87 and 2.12 (*p* < 0.0001) in 276 HER2-amplified breast cancer patient cohort (Fig. 1D) indicating potential prognostic role of DVL2 in HER2+ breast cancer patients.

### DVL2 is present in the nucleus of HER2+ breast cancer cells and regulates transcription of key immune-modulatory genes

**Fig. 2:**
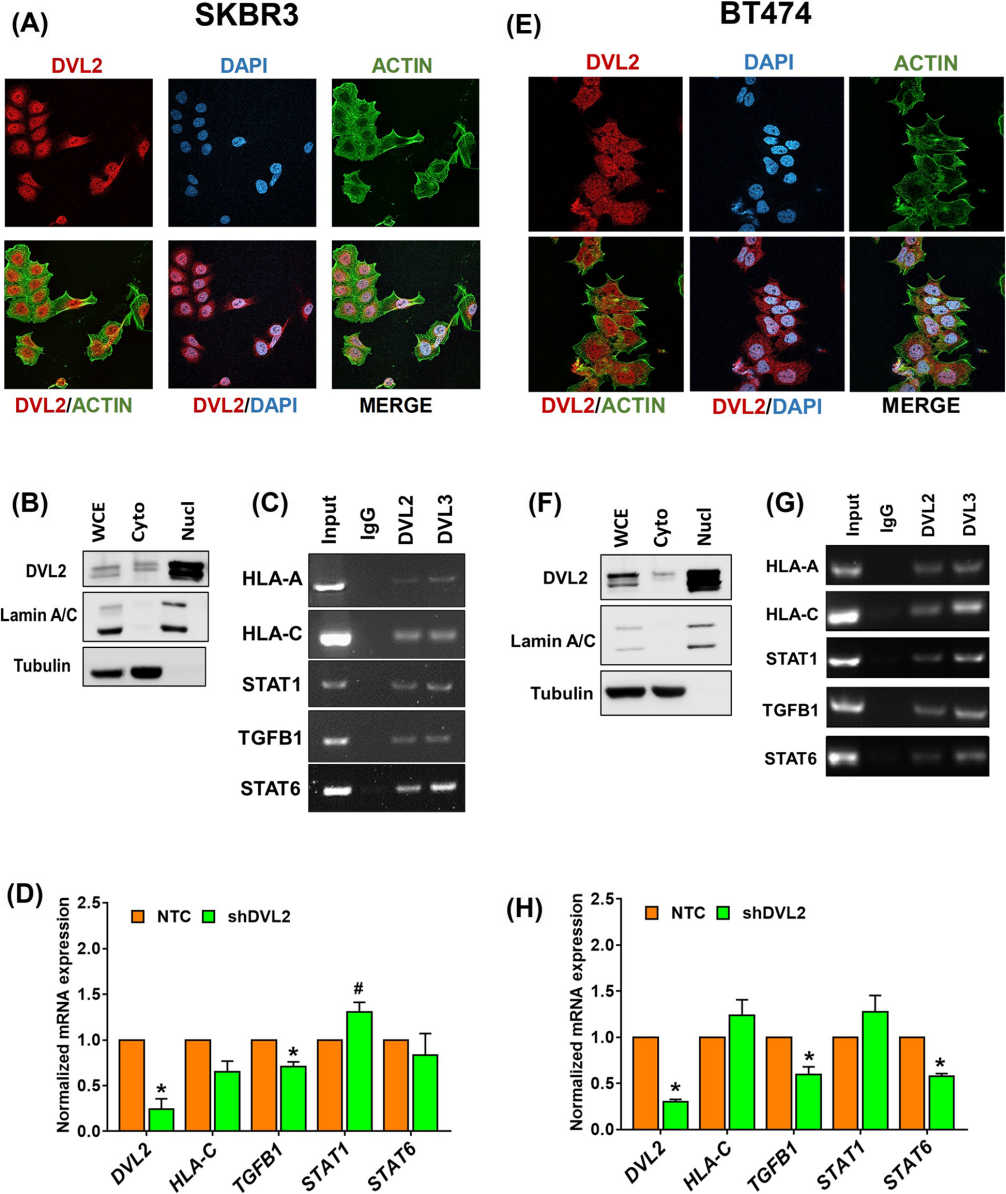
Nuclear DVL2 binds to promoter regions and regulates transcription of infiltrating T-cell genes in HER2+ breast cancer cell lines. **A** Immunofluorescence staining was performed to analyze DVL2 proteins localization in SKBR3 (ER−/PR−/HER2+) breast cancer cells. The cells were probed with DVL2 (red). The nucleus was stained with DAPI (blue) and the actin filaments (green) were stained with Phalloidin. **B** Whole (WCE), cytoplasmic (Cyto) and nuclear (Nucl) extracts from SKBR3 breast cancer cells were analyzed using western blots. The blots were probed with DVL2 antibody. Lamin was used as a control for nuclear extract and Tubulin was used as a control for cytosolic proteins. **C** ChIP PCR was performed for different immune-modulatory genes for Input, IgG, DVL2 and DVL3 in SKBR3 breast cancer cells. **D** RT-qPCR based mRNA expression analyses of *DVL2, HLA-C, STAT1, TGFB1, STAT6* in SKBR3 cells stably expressing NTC vs. shDVL2. Transcript levels were normalized using *GAPDH*, *B2M* and *GUSB* as housekeeping control. All experiments were performed in triplicates and Welch’s independent T-test were performed between NTC and shDVL2 where *p < 0.05 and ^#^*p* < 0.1. **E** Immunofluorescence staining was performed to analyze DVL2 proteins localization in BT474 (ER+/PR+/HER2+) breast cancer cells. The cells were probed with DVL2 (red). The nucleus was stained with DAPI (blue) and the actin filaments (green) were stained with Phalloidin. **F** Whole (WCE), cytoplasmic (Cyto) and nuclear (Nucl) from BT474 breast cancer cells were analyzed using western blots. The blots were probed with DVL2 antibody. Lamin was used as a control for nuclear extract and Tubulin was used as a control for cytosolic proteins. **G** ChIP PCR was performed for different immune-modulatory genes for Input, IgG, DVL2 and DVL3 in BT474 breast cancer cells. **H** RT-qPCR based mRNA expression analyses of *DVL2, HLA-C, STAT1, TGFB1, STAT6* in BT474 cells stably expressing NTC vs. shDVL2. Transcript levels were normalized using *GAPDH*, *B2M* and *GUSB* as housekeeping control. All experiments were performed in triplicates and Welch’s independent T-test were performed between NTC and shDVL2 where *p < 0.05 and ^#^*p* < 0.1

Previously we reported that DVL proteins are present in the nucleus of TNBC cells and modulates various genes expression involved in tumor biology, metabolism, and immunity [27]. Here, we investigated the effect of nuclear DVL2 in top immune-modulatory genes from our published DVL3-ChIP-seq analyses in TNBC. We identified DVL2 nuclear localization via immune staining and sub-cellular fractionation in two different HER2+ breast cancer cell lines (Fig. 2A-B & E-F). Next, we determined whether DVL2 binds to the promoter regions of genes involved in antigen presentation (*HLA-A, HLA-C*) and T-cell maintenance (*STAT1, STAT6, TGFB1*) in HER2-positive breast cancer cells. Notably, we found both DVL2 and DVL3 binds to the promoter regions of *HLA-A, HLA-C, STAT1, TGFB1, STAT6* (Fig. 2C, G) and DVL2 loss of function results in decreased *TGFB1* mRNA transcription (NTC vs. shDVL2; *p* < 0.05) in both SKBR3 and BT474 cell lines. In addition, with DVL2 knockdown, there was a trend of increase in *STAT1* mRNA expression levels (*p* < 0.1) in SKBR3 cells while significant reduction in *STAT6* mRNA levels were observed in BT474 cells (*p* < 0.05) (Fig. 2D, H). Of note, both TGFB1 and STAT6 are well-known for their immune evasive roles in breast tumor microenvironment while STAT1 is proposed as a potential biomarker in breast cancer patients undergoing immune checkpoint therapy [34–36]. In contrast, we didn’t observe any significant increase in *HLA-C* mRNA levels and *HLA-A* mRNA expression levels were unidentifiable in both in vitro models of HER2+ breast cancer. Therefore, these findings indicate promising immune-regulatory role of DVL2 in HER2+ breast cancer cells.

### DVL2 loss of function results in reduced expression of Wnt target genes, reduction in cell proliferation and cell cycle progression in HER2+ breast cancer cells with in vitro HER2 inhibition

**Fig. 3:**
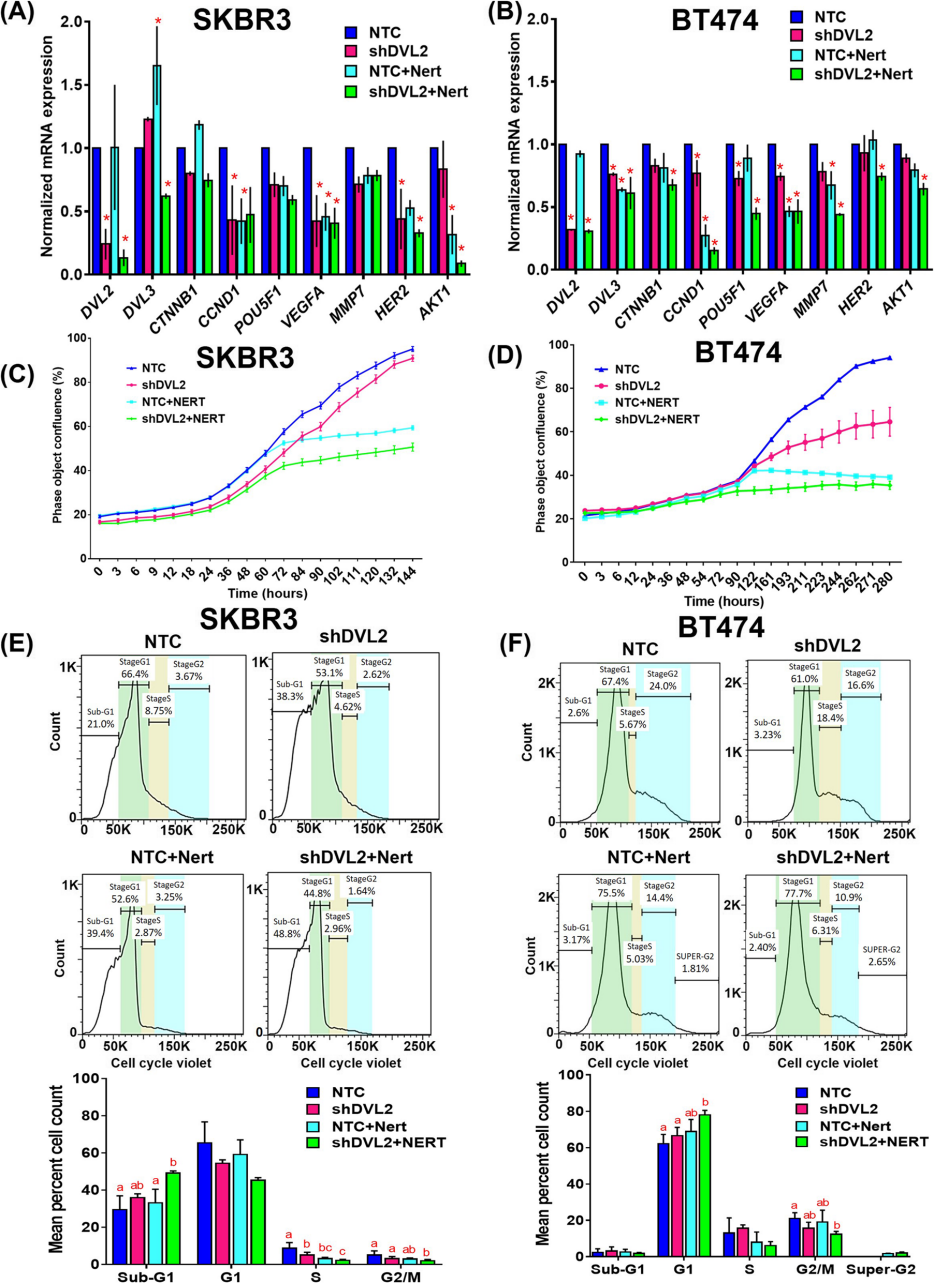
DVL2 loss of function reduces in vitro Wnt target genes expression, cell proliferation and cell cycle progression ± HER2 inhibition. **A**, **B** RT-qPCR based mRNA expression analyses of Wnt target genes (*DVL2, DVL3, CTNNB1, CCND1, POU5F1, VEGFA, MMP7, ERBB2, AKT1*) respectively in SKBR3 and BT474 cells stably expressing NTC vs. shDVL2 with or without 1 nM Neratinib (Nert) treatments. Transcript levels were normalized using *GAPDH*, *B2M* and *GUSB* as housekeeping control. All experiments were performed in triplicates and Welch’s independent T-test were performed between NTC vs. shDVL2 or NTC vs. NTC + Nert or NTC + Nert vs. shDVL2 + Nert to investigate the additive effect of HER2 inhibition with DVL2 loss of function (*n* = 3; *p < 0.05). **C**, **D** 2D proliferation assay was performed in SKBR3 and BT474 breast cancer cell lines via in vitro live cell image analyses. SKBR3 and BT474 cells stably expressing NTC vs. shDVL2 were seeded in a 96-well plate and upon confluence were treated with 1 nM Nert or DMSO vehicle. Cells were monitored and live cell percentages were quantified in real time via IncuCyte ZOOM (Essen Biosciences) for 144 and 280 h respectively for SKBR3 and BT474 cells. For each time points Mean ± SEM values were shown and considered statistically significant at *p < 0.05. **E**, **F** The effect of HER2 inhibition via ± 1 nM Neratinib (Nert) treatment respectively in SKBR3 and BT474 cells stably expressing NTC vs. shDVL2 was analyzed in a cell cycle assay using a Vybrant cell violet dye (Invitrogen) followed by flow cytometry. The live cells are represented in the SSC vs. FSC plots, which were further distributed among different cell cycle stages such as SubG1, G1, S, and G2/M phases. Experiments were performed in triplicates and mean values were shown in the graphs below. One-way Anova with Tukey’s multiple comparison test were performed where different letters denote statistical significance at *p* < 0.05

DVL2 is critical in regulating various cellular processes [37] and silencing of DVL2 results in reduced cancer cell proliferation, migration, and invasiveness [38–40]. Hence, we examined the effects of DVL2 loss of function in the transcription of multiple key Wnt target genes involved in cell proliferation, stem cell renewal, angiogenesis, cell migration and invasion with or without HER2 inhibition in vitro. We utilized two different HER2-amplified breast cancer cell lines stably expressing NTC vs. shDVL2 and treated them with 1 nM neratinib or vehicle control. Reduction in DVL2 function via shRNA resulted in reduced mRNA expression of *CCND1* and *VEGFA*, genes which are involved in cancer cell proliferation and angiogenesis respectively in both SKBR3 and BT474 cells (*p* < 0.05). Additionally, mRNA expression of *POU5F1*, marker of stem cell renewal, was reduced significantly in BT474 cells expressing NTC vs. shDVL2 (*p* < 0.05). Interestingly, mRNA expression of other genes involved in cancer invasiveness such as *DVL3*, *CTNNB1, MMP7, HER2, AKT1* were not downregulated by shDVL2 alone compared to NTC but reduced in combination of neratinib with shDVL2 compared to NTC + Nert in either or both SKBR3 and BT474 cell lines (*p* < 0.05) (Fig. 3A-B) indicating promising additive anti-cancer effects of neratinib and DVL2 silencing in HER2+ breast cancer. To further investigate this phenomenon, we performed in vitro live cell proliferation assays with or without neratinib treatment in SKBR3 and BT474 cells expressing NTC vs. shDVL2. We noted significant reduction in percent live cell confluence with shDVL2 (vs. NTC) at 24–102 h and shDVL2 + Nert (vs. NTC + Nert) after 72 h in SKBR3 cells (*p* < 0.05) as well as at 122 h and onwards in BT474 cells between shDVL2 and shDVL2 + Nert treatments (*p* < 0.05) (Fig. 3C-D). Although reduction in cell confluence and/or proliferation was not significant between NTC vs. shDVL2 in SKBR3 and between NTC + Nert vs. shDVL2 + Nert in BT474 till the end of the experimental time points, continual decrease in cell proliferation observed in shDVL2 + Nert vs. shDVL2 alone in both cell lines indicating possible additive effect against cancer cell proliferation with the combination of HER2 and DVL2 inhibition. Next, to determine the individual and/or combined effects of DVL2 and HER2 inhibition on cell-cycle phases in HER2-amplified breast cancer cell lines, we studied the different stages in actively proliferating (live) SKBR3 and BT474 cells stably expressing NTC vs. shDVL2 and treated them with 1 nM Nert or DMSO control (Fig. 3E-F). The cells were stained with Vybrant cell violet dye and distributed in four cell cycle phases namely, (i) Gap 1 (G1), (ii) DNA synthesis (S), (iii) cell growth and mitosis (G2/M) and (iv) growth or G1-arrest (Sub-G1) stages via flow cytometry. In SKBR3 cells, we observed a significant increase in the cell population undergoing growth-arrest (Sub-G1) with shDVL2 + Nert combination compared to NTC + Nert and shDVL2 alone (*p* < 0.05) while there was a significant reduction in cell population in S and G2/M phases with shDVL2 and Nert combination compared to either shDVL2 alone or Nert alone (NTC + Nert) (*p* < 0.05). Similarly in BT474 cells, there was a significant increase in cell population in G1 phase with shDVL2 + Nert combination compared to NTC and shDVL2 alone while significant reduction was observed in G2/M phase population with shDVL2 + Nert combination compared to NTC control (*p* < 0.05) indicating that the additive effect of DVL2 and HER2 inhibition against cancer cell proliferation might be more prominent in HER2-amplified cells which are hormone receptor negative not hormone receptor positive (ER/PR+) cells. Findings from these studies clearly demonstrate the promising combinatorial anti-proliferative role of DVL2 and HER2 silencing in HER2+ breast cancer.

### DVL2 loss of function modulates EGFR signaling cascade in HER2+ breast cancer cells with or without in vitro HER2 inhibition

**Fig. 4:**
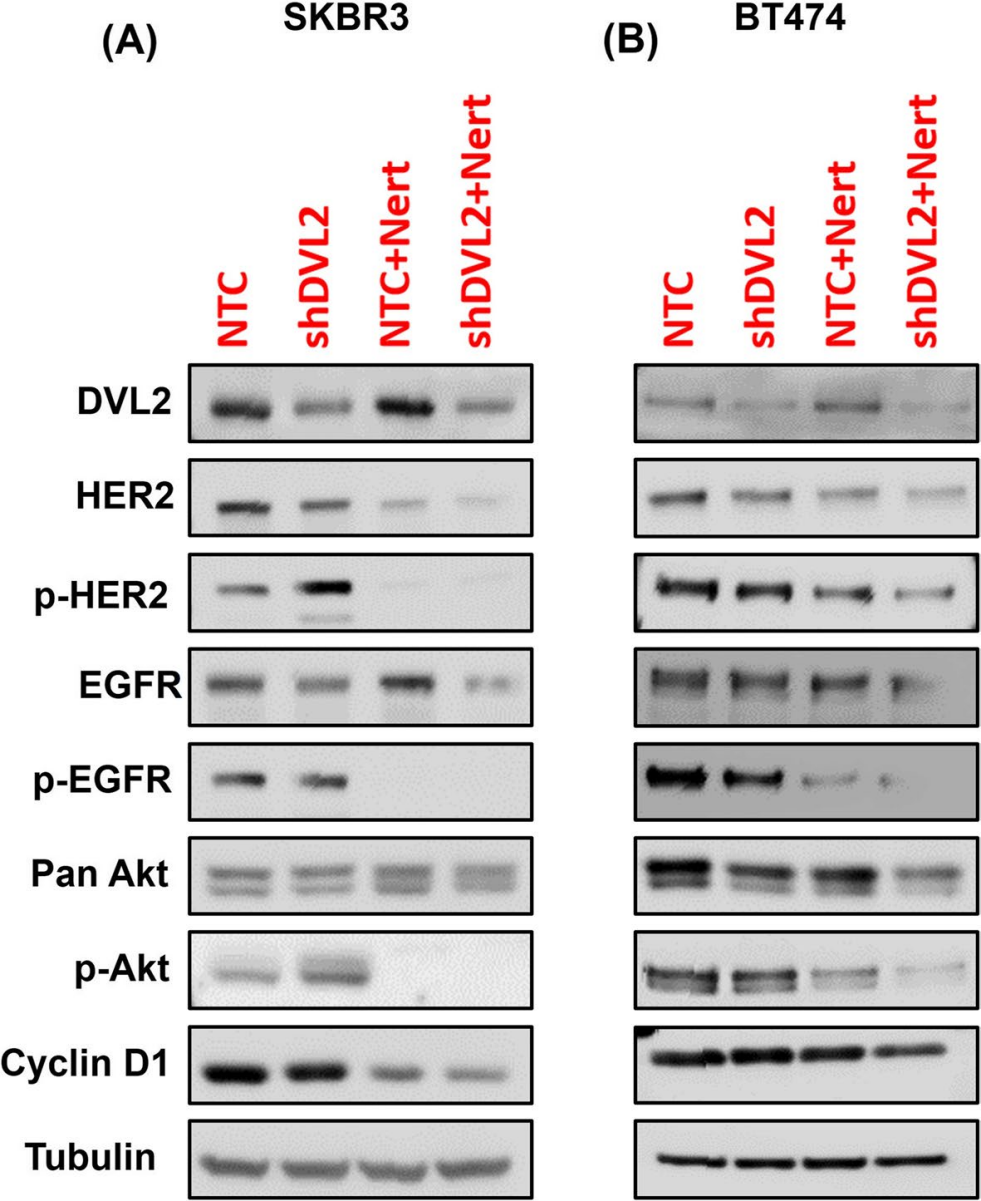
DVL2 depletion modulates Wnt and HER2 signaling cascades with or without in vitro HER2 inhibition. **A**, **B** Immunoblot analyses of lysates respectively from SKBR3 and BT474 cells stably expressing NTC vs. shDVL2 and treated with 1 nM neratinib (Nert) or DMSO control for 24 h and probed with antibodies as indicated with Tubulin as housekeeping control. Please also see Methods and Supplementary Table 1 for additional information

Previous literatures indicate critical role between Wnt and EGFR signaling convergence in cancer progression [41, 42], yet handful of them investigated the mechanistic role of dishevelled proteins in regulating EGFR signaling in breast cancer [43, 44] and none reported the mechanistic and functional effects of DVL2 modulation in HER2+ breast cancer. In this section, we aim to critically investigate the role of DVL2 depletion in regulating key proteins of EGFR signaling cascade with or without HER2 inhibition in two different in vitro models of HER2+ breast cancer. For this purpose, we treated SKBR3 and BT474 HER2-amplified cell lines stably expressing NTC vs. shDVL2 with 1 nM neratinib (Nert) or DMSO control for 24 h and performed western blot analyses. We observed significant reduction in DVL2 protein expression in response to shDVL2 + Nert treatment compared to NTC + Nert and shDVL2 alone in both SKBR3 and BT474 cells (Fig. 4A-B, Supplementary Table 1). In addition, there was a marked decrease in phospho-HER2, total HER2, phospho-EGFR, total EGFR, phospho-AKT, total AKT and cyclin D1 protein expression with shDVL2 + Nert treated BT474 cells compared to shDVL2 alone and Nert (NTC + Nert) alone. Similar results were found in all proteins expression in SKBR3 cells when treated with shDVL2 + Nert compared to shDVL2 alone (Fig. 4A-B, Supplementary Table 1). Interestingly, increased protein expression for phospho-HER2 and phosphor-AKT were observed in shDVL2 groups while increased DVL2, total EGFR and total AKT expression were found in NTC + Nert treated groups compared to other treatments in SKBR3 cells (Supplementary Table 1). This result indicates a possible DVL2-centric feedback loop between EGFR and non-canonical Wnt signaling which needs further investigation. In summary, results from these experiments reports a novel mechanistic role of DVL2 and HER2 depletion in modulating EGFR signaling activation in HER2+ breast cancer, which in turn, opens a new area of investigation of how Wnt and EGFR crosstalk might affect HER2+ breast cancer progression in the clinical setting.

### Mapping of DVL2 expression in a HER2+ breast cancer cohort reveals promising correlation with clinical predictors of breast cancer survival

**Fig. 5:**
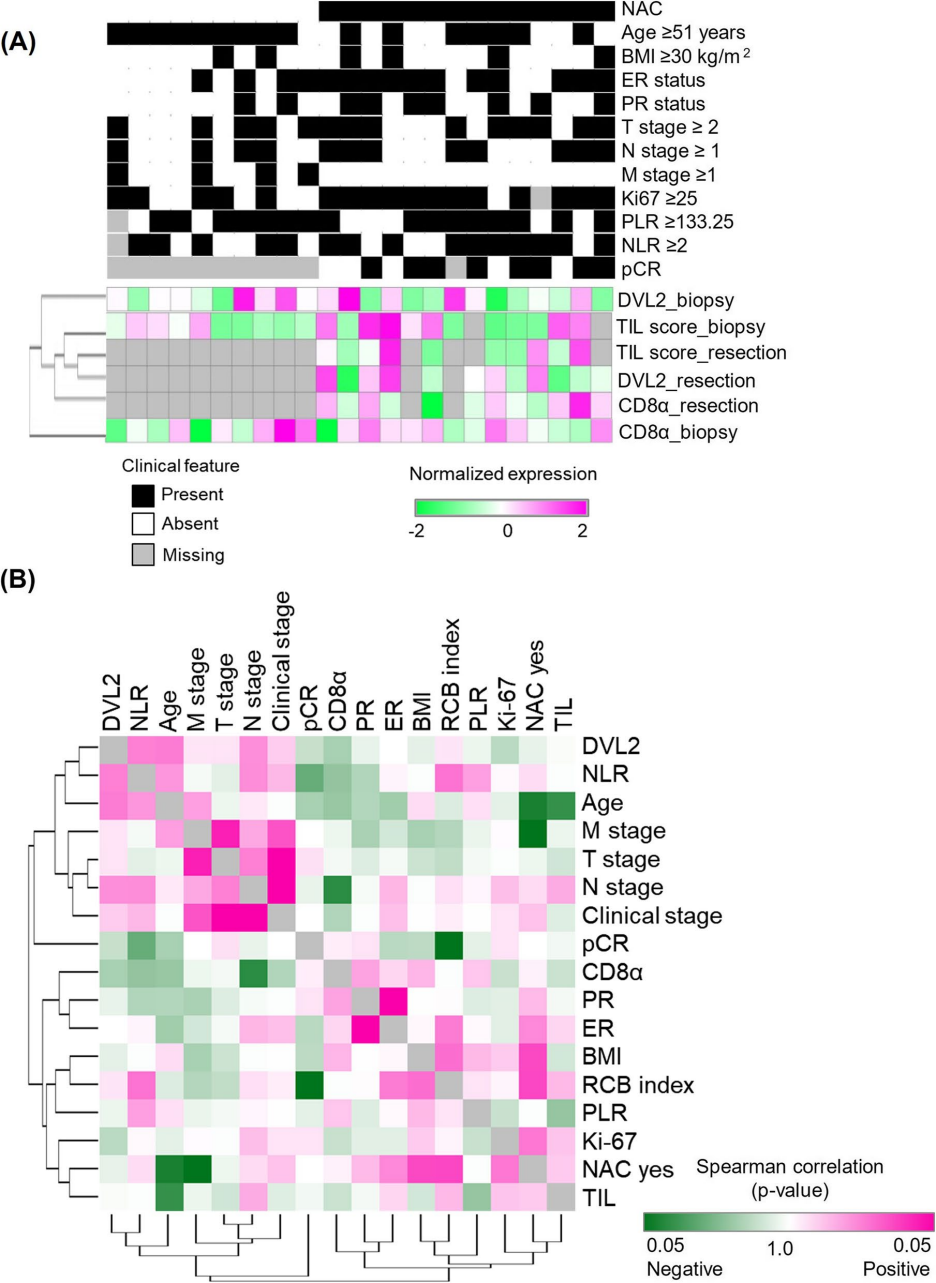
Mapping of DVL2 with clinical predictors of survival in a HER2-positive breast cancer patient cohort. **A** Heatmap showing expression patterns of DVL2, TIL-score and CD8α in association with clinical parameters of breast cancer at biopsy and after resection in 24 HER2+ breast cancer patients who received NAC (*n* = 14) vs. no NAC (*n* = 10). **B** Correlation between DVL2, CD8α and multiple clinical prognostic predictors (TIL, NLR, PLR, pCR, RCB index) and/or clinical demographics of breast cancer in 24 HER2+ breast cancer patients at baseline were evaluated using Spearman’s rank correlation test and graph was generated using Morpheus software (https://software.broadinstitute.org/morpheus). TIL: Tumor infiltrating lymphocytes; NLR: Neutrophil to lymphocyte ratio; PLR: Platelet to lymphocyte ratio; pCR: Pathologic complete response; RCBI: Residual cancer burden index

To further validate our in vitro analyses of DVL2 role in HER2+ breast cancer, we performed TIL-scoring and mapped DVL2 and CD8α protein levels via immune staining in a preliminary sample of 24 HER2+ breast cancer patients at baseline biopsy (*N* = 24) and post NAC resection (*N* = 14) tissues. Figure 5A and Supplementary Table 2 describes distribution of patient characteristics in association with DVL2 expression at baseline where higher DVL2 expression was significantly associated with higher age group patients who were 51 years or older (*N* = 16, *p* = 0.039) and trend of association was observed between high DVL2 and lymph node (N) stage 1 or more (*N* = 12, *p* = 0.074). Additionally, paired analyses of DVL2 expression and TIL score in biopsy and resection tissue samples revealed significant inverse association between baseline DVL2 and TIL score (*N* = 24, *p* < 0.05) while a trend of association was observed between DVL2 and TIL score post resection (*n* = 10, *p* = 0.063). Similarly, baseline DVL2 and CD8α levels were significantly associated in biopsy tissues (*N* = 24, *p* < 0.0001) but no significant association was observed between the two protein markers after resection (Supplementary Table 3). Next, we performed Spearman’s rank correlation analyses between DVL2 expression and multiple clinical and prognostic indicator of breast cancer survival. While DVL2 expression in baseline biopsy tissues didn’t significantly correlate with recorded clinic-pathological variables of breast cancer, a trend of significant positive correlations was observed between DVL2 and multiple clinical markers of high-risk breast cancer namely age, −stage>N1, NLR (Fig. 5B, Supplementary Table 4). Further subgroup analyses revealed a significant negative correlation between baseline TIL score and age (*p* < 0.05) as well as baseline CD8α levels and N-stage> 1 (*p* < 0.05) (Supplementary Table 4) denoting possible association between DVL2 and immune-regulatory markers of HER2+ breast cancer.

**Fig. 6:**
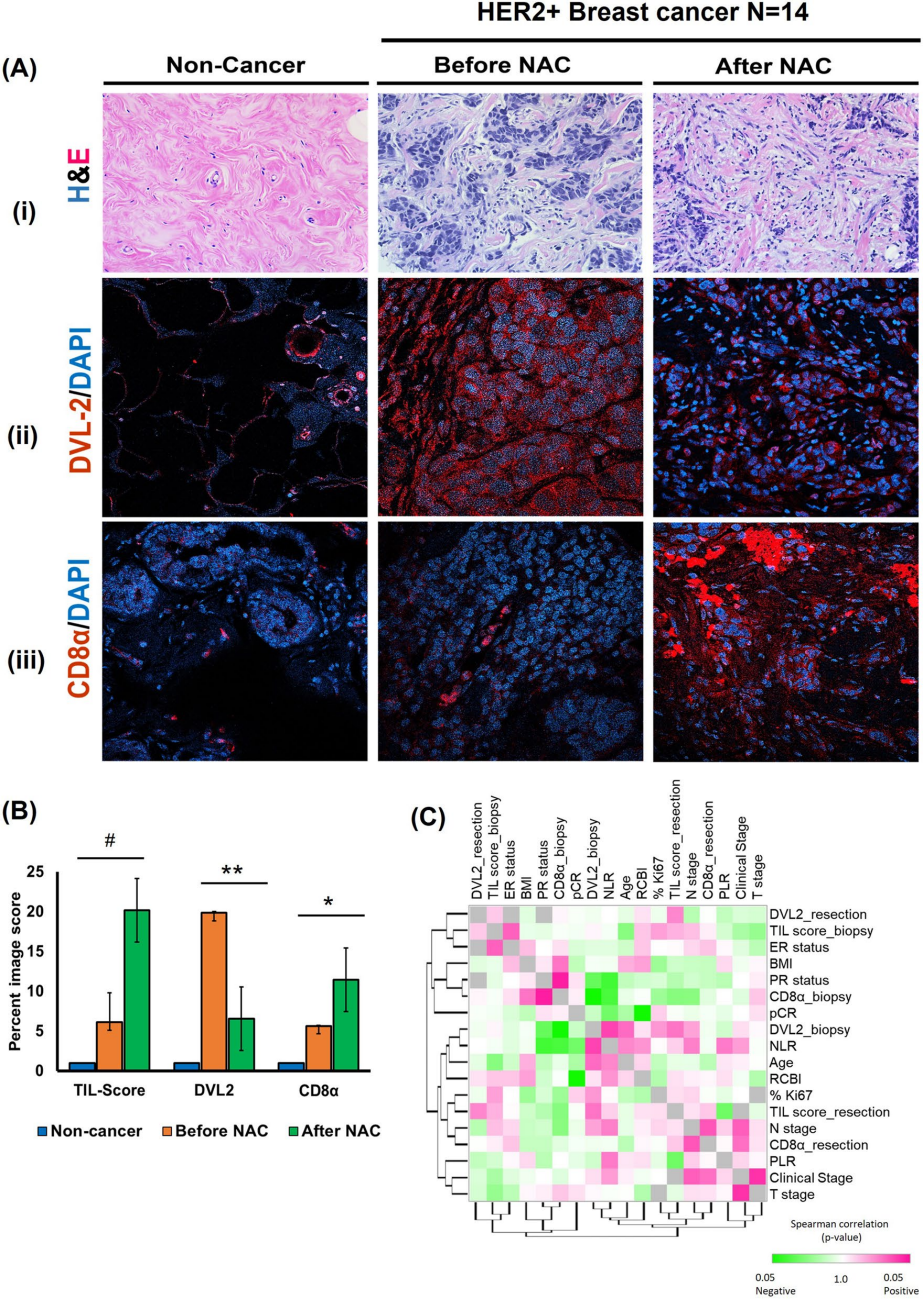
TIL scoring and phenotyping in association with DVL2 and CD8α levels in a HER2-positive breast cancer patient cohort before (biopsy) and after (resection) NAC. **A** Data shown are for benign tissue and breast cancer specimens before NAC (biopsy) and after NAC (resection); (i) Hemoxylin and Eosin staining for TIL score analysis, (ii) DVL2 (red) and DAPI (blue) immunofluorescent staining, and (iii) CD8α (red) and DAPI (blue) immunofluorescent staining. **B** Group mean percent image score with SD plotted against TIL-scoring and prevalence normalized fluorescence for each antibody marker before NAC (biopsy) and after NAC (resection) specimens (*N* = 14; **p < 0.001; *p < 0.05; #*p* < 0.1). **C** Correlation between DVL2, CD8α and multiple clinical prognostic predictors (TIL, NLR, PLR, pCR, RCB index) and/or clinical demographics of breast cancer in 14 HER2-positive breast cancer patients who received NAC. Normalized expression levels for each variable after biopsy were evaluated using Spearman’s rank correlation test and graph was generated using Morpheus software (https://software.broadinstitute.org/morpheus). TIL: Tumor infiltrating lymphocytes; NLR: Neutrophil to lymphocyte ratio; PLR: Platelet to lymphocyte ratio; pCR: Pathologic complete response; RCBI: Residual cancer burden index. UALCAN: The University of ALabama at Birmingham CANcer data analysis Portal

Next, to further investigate the immunoregulatory and prognostic role of DVL2 in response to NAC, we performed a subgroup analysis between DVL2 and different clinical and/or prognostic markers of breast cancer in biopsy and resection specimens of patients who received NAC. Among 24 HER2+ breast cancer patients, 14 patients received standard NAC and of them, 7 patients attained complete pathological response (pCR) while pCR data was unavailable for 2 patients and remaining 5 patients had residual tumor with RCBI I or II. Figure 6A shows the representative images of TILs, DVL2 and CD8α levels at baseline biopsy (before NAC) and post resection (after NAC) specimens where we observed a significant reduction in DVL2 expression (*p* < 0.05) and a significant increase in CD8α levels (*p* < 0.05) in after NAC specimens compared to before NAC specimens after quantification of respective proteins in 14 HER2+ patients (Fig. 6B). Though not statistically significant, we did detect a trend of increased TIL scores (*p* < 0.1) in post resection samples compared to biopsy samples which designates the efficacy of NAC in this patient cohort (Fig. 6B). In addition to the promising staining results, we examined the correlation between DVL2 and prominent clinical predictors of breast cancer in patients who received NAC (before and after) (Fig. 6C). Interestingly, we found a significant positive correlation between baseline DVL2 levels and NLR (rho = 0.58; *p* = 0.033), which is a poor prognostic indicator of breast cancer survival while there was a negative correlation between DVL2 and cytotoxic T-cell marker, CD8α at baseline (rho = − 0.67; *p* = 0.010). Additionally, CD8α levels were negatively correlated with NLR (rho = − 0.59; *p* = 0.030) denoting probable crosstalk between DVL2 and CD8α proteins in predicting prognostic outcomes of HER2+ breast cancer (Fig. 6C, Supplementary Table 5). Of note, there was a trend of significant positive correlation between baseline DVL2 and TIL score post NAC, and a trend of significant negative correlation between baseline CD8α and post NAC TIL score (Supplementary Table 6) depicting complex interaction between DVL2 and immune cell population in response to NAC in HER2+ breast cancer which requires more detailed investigation.

While we identified some novel thought-provoking associations between DVL2 and multiple clinical prognostic indicators of breast cancer survival, we failed to observe these correlations in HER2+ patients who didn’t receive NAC (Supplementary Table 7). This reveals another aspect that DVL2 might act as a predictor of prognostic biomarkers specifically in aggressive HER2+ breast cancer which would require further future analysis in a larger cohort of breast cancer patients.

## Discussion

DVL is a key mediator of Wnt signaling pathway and is involved in the development and signal transduction [33, 45]. Wnt signaling mediates cell proliferation, asymmetric cell division, cell fate determination, stem cell maintenance, and cell polarity [46]. Given its significance in many contexts, its deregulation is associated with various diseases including cancer. Hence, in our study we reported a novel role of DVL2 in oncogenic Wnt and EGFR signaling modulation as well as its promising correlation with markers of tumor immune regulation (such as TILs and cytotoxic CD8α).

Recently, β-catenin signaling was shown to promote immune suppression in patients treated with immune checkpoint blockade therapies [21, 47, 48]. Other reports demonstrated that β-catenin-independent Wnt signaling also contributes to immune suppression and aberrant secretion of Wnt antagonists such as Dickkopf1 by tumor cells impede the function of cytotoxic lymphocytes and natural killer (NK) cells in the TME [49]. However, an understanding of the extent to which proteins upstream of β-catenin are involved in regulating lymphocyte infiltration remains mostly unknown in the field. Given the role of DVL as a critical activator of β-catenin-dependent and -independent Wnt signaling, it is important to investigate potential relationships between DVL and the regulation of TIL recruitment. Our findings provide support for the need to further investigate the role of DVL in regulating TIL dynamics. While it is well known that DVL transmits signals from aberrantly expressed Wnts, virtually nothing is known about the role of DVL paralogs in mediating tumor cell intrinsic regulation of immune cells. This question is particularly important considering ongoing clinical trials investigating the role of multiple Wnt pathway inhibitors (small molecules and anti-Frizzled antibodies) as novel anti-cancer approaches [50–52]. Our previous findings provided clues regarding potential mechanisms by which DVL may regulate TIL dynamics. We previously found that many of the novel DVL targets bound by DVL proteins are involved in cell migration, antigen presentation, immune surveillance and chemoattraction [27, 29]. Our bioinformatics analysis of DVL binding patterns identified a statistically significant enrichment of genomic sequences with a significant enrichment of transcription factor (TF) consensus sequences for key TFs such as ZBTB7B/ThPOK and STAT3, which have been shown to regulate immune function [53] and mammary gland function [54]. ZBTB7B, is a transcriptional repressor and is shown to play a role in regulating maturation of T lymphocytes. The integration of published ZBTB7B chip seq data with our previously published DVL3 chip seq data [27] revealed that majority of chip hits between ZBTB7B and DVL3 are linked with immune system and lymphocyte maturation. Our current and previous findings suggest that one or more DVL paralogs whose expression is dysregulated in tumor cells may be involved in regulating genes that influence TIL recruitment to the TME [27, 31].

In this study, we used the UALCAN analysis to emphasize the differential expression of DVL2 in different molecular subtypes of breast cancer and observed that DVL2 is upregulated in the breast tumor samples compared to the benign breast tissues. Immunofluorescent staining revealed that DVL2 exhibited higher expression in HER2+ breast cancer tissues compared to the adjacent tissues and Kaplan Meier survival curves analysis revealed that increased DVL2 expression was associated with poor overall survival, hence poor prognosis in HER2+ breast cancer patients. The human immune system is usually capable of destroying the tumor by cancer immunity cycle. However, some tumors escape this cycle through various factors and our findings suggested that DVL2 might be one of them that aid the cancer cells in suppressing and evading the immune response. Herein, we investigated the DVL2 association with genes involved in antigen presentation and T cell maintenance in HER2+ breast cancer cell lines. Our Chip PCR demonstrated that DVL2 localizes to the immune related genes. Additionally, mRNA expression analysis demonstrated that knockdown of DVL2 resulted in upregulation of STAT1, suggesting that DVL2 acts as a negative regulator of tumor suppression.

While previous studies show a role of DVL2 silencing in proliferation in vitro, our report herein for the first time showed the depletion of DVL2 along with in vitro HER2 inhibition by neratinib, clinically approved HER2 inhibitor, shows an additive anti-cancer effect in two HER2-overexpressed breast cancer cell lines, BT474 and SKBR3. Moreover, we observed distinct differences in mRNA expression of cancer associated genes with DVL2 and HER2 inhibition. First, we observed that DVL2 directly regulates expression of genes involved in cancer hall marks, CCND1, VEGFA and POU5F1. Second, we found that depletion of DVL2 along with HER2 has an additive inhibitory effect on genes involved in cancer invasion. The combination also suppressed cell proliferation in both cell lines. The cell cycle analysis revealed that the combination of shDVL2 and Neratinib affects the cell growth in different stages of cell cycle when compared to the individual treatment. Neratinib irreversibly binds to ERBB1/2/4 and inhibits cell growth inducing autophagy and mitochondrial dysfunction [55]. Considering that the mode of cell death influences the anti-tumor immune response and since DVL2 affects cell growth, further investigation is required to determine the mechanism by which shDVL2 enhances Neratinib in mediating cell death and its influence on the immunogenicity. These results will provide new insights in developing a combinatorial treatment option in treating HER2+ breast cancer.

Previous studies reported that Wnt and EGFR plays key role in embryonic development and cell proliferation and the crosstalk between two pathways often leads to tumorigenesis with poor prognosis [41, 56]. The present study uncovers the link between the DVL proteins and HER2 receptors in regulating proteins of EGFR signaling pathway. Using the protein expression analysis, we identified that HER2, EGFR, AKT, cyclin D1 protein expression was significantly lower in the combination therapy when compared to the individual treatments. This might suggest a possibility that Wnt and EGFR crosstalk effects the regulation of HER2+ breast cancer.

The present study reports DVL2 association with CD8α levels in HER2+ breast cancer biopsy tissues. NAC is frequently used for early and advanced stage breast cancer patients, and our study demonstrates that high DVL2 is found in biopsy tissue before the NAC and high CD8α levels are found in biopsy tissues post NAC. Similarly, DVL2 positively correlates with NLR, while a negative correlation was found between DVL2 and CD8α, and in between CD8α and post NAC TIL score, suggesting an association between DVL2 and TIL score in HER2+ breast cancer.

In summary, this report examines the novel link between DVL2 and TILs in HER2+ breast cancer. DVL2 expression is negatively correlated with the presence of cytotoxic immune cells in the TME suggesting that DVL2 might serve as a prognostic biomarker in HER2+ breast cancer. Our study is noteworthy since examining the crosstalk between the Wnt and HER2 pathways presents novel insight into the possible immune regulatory role of DVL2 proteins in HER2+ breast cancer. This, in turn, can be used in designing future early-phase clinical trials and in-depth translational studies for optimizing treatment targeting Wnt/HER2 mediated immune-regulation in different subtypes of breast cancer. A deeper understanding of DVL2 biology in HER2+ tumors might lead to the development of novel therapeutic strategies not previously imagined.

## Supplementary Information

**Additional file 1: Figure S1a.** Western blot analysis showing the knockdown of DVL2. **Figure S1b.** Raw data for western blots in Fig. S1a. **Figure S2a.** Raw data for western blots in Fig. 2. **Figure S2a.** Raw data for ChIPdatain Fig. 2. **Figure S2b.** Raw data for ChIPdatain Fig. 2. **Figure S4a.** Raw data for western blots in Fig. 4. **Figure S4b.** Raw data for western blots in Fig. 4.

**Additional file 2: Table S1.** Protein quantification of western blots of SKBR3 and BT474 cells expressing NTC vs shDVL2 treated with Neratinib. **Table S2.** Association between DVL-2 protein expression and clinicopathological parameters in HER2-positive breast cancer patient cohort at baseline (*N* = 24,*p < 0.05). **Table S3.** Association between DVL-2 protein expression and cancer survival predictors in HER2-positive breast cancer patient cohort (*N* = 24, *p* < 0.05). **Table S4.** Spearman’s rank correlation matrix for DVL2 and clinical parameters and/or survival predictors of breast cancer in 24 HER2-positive breast cancer patients after biopsy at baseline. **Table S5.** Spearman’s rank correlation matrix for DVL2 and clinical parameters and/or survival predictors of breast cancer at baseline in 14 HER2-positive breast cancer patients who received neoadjuvant chemotherapy (NAC). **Table S6.** Spearman’s rank correlation matrix for DVL2 and clinical parameters and/or survival predictors of breast cancer after resection in 14 HER2-positive breast cancer patients who received neoadjuvant chemotherapy (NAC). **Table S7.** Spearman’s rank correlation matrix for DVL2 and clinical parameters and/or survival predictors of breast cancer at baseline biopsy in 10 HER2-positive breast cancer patients who didn’t receive neoadjuvant chemotherapy (NAC). **Table S8.** ChIP primers used for the study. **Table S9.** mRNA Primers used for the study. **Table S10.** Antibodies used for the study.

## Data Availability

Results in this manuscript were in part based upon data generated by the TCGA Research Network: https://portal.gdc.cancer.gov/. Additional data used in this publication were generated by the Clinical Proteomic Tumor Analysis Consortium (NCI/NIH) - CPTAC Data Portal: https://proteomics.cancer.gov/data-portal. The datasets generated and/or analyzed during the current study are available in the Gene Expression Omnibus (GSE165774) https://www.ncbi.nlm.nih.gov/geo/query/acc.cgi?acc=GSE165774. All data needed to evaluate the conclusions in the paper are present in the manuscript and the Supplementary Materials. Additional data is available upon request from the corresponding author.

## Abbreviations

DVL: Dishevelled
BMI: Body mass index
ER: Estrogen receptor
HER2: Human epidermal growth factor receptor 2
NLR: Neutrophil lymphocyte ratio
NFκB: Nuclear factor kappa B
pCR: Pathological Complete Response
PLR: Platelet Lymphocyte Ratio
RCBI: Residual Cancer Burden Index
TCGA: The Cancer Genome Atlas
TILs: Tumor infiltrating lymphocytes
TNBC: Triple-negative breast cancer
NAC: Neoadjuvant chemotherapy
