# Supplementary material for: Dishevelled 2 regulates cancer cell proliferation and T cell mediated immunity in HER2-positive breast cancer
Source: BMC Cancer. 2023 Feb 21;23:172. doi: 10.1186/s12885-023-10647-2 (PMC9942370; doi:10.1186/s12885-023-10647-2)
Supplement: Supplementary file 1 — Additional file 1: Figure S1a. Western blot analysis showing the knockdown of DVL2. Figure S1b. Raw data for western blots in Fig. S1a. Figure S2a. Raw data for western blots in Fig. 2. Figure S2a. Raw data for ChIPdatain Fig. 2. Figure S2b. Raw data for ChIPdatain Fig. 2. Figure S4a. Raw data for western blots in Fig. 4. Figure S4b. Raw data for western blots in Fig. 4. [file 12885_2023_10647_MOESM1_ESM.pdf]

Dishevelled 2 regulates cancer cell proliferation and T cell mediated immunity in HER2-positive breast cancer

Supplementary figures

Figure S1a - Western blot analysis showing the knockdown of DVL2

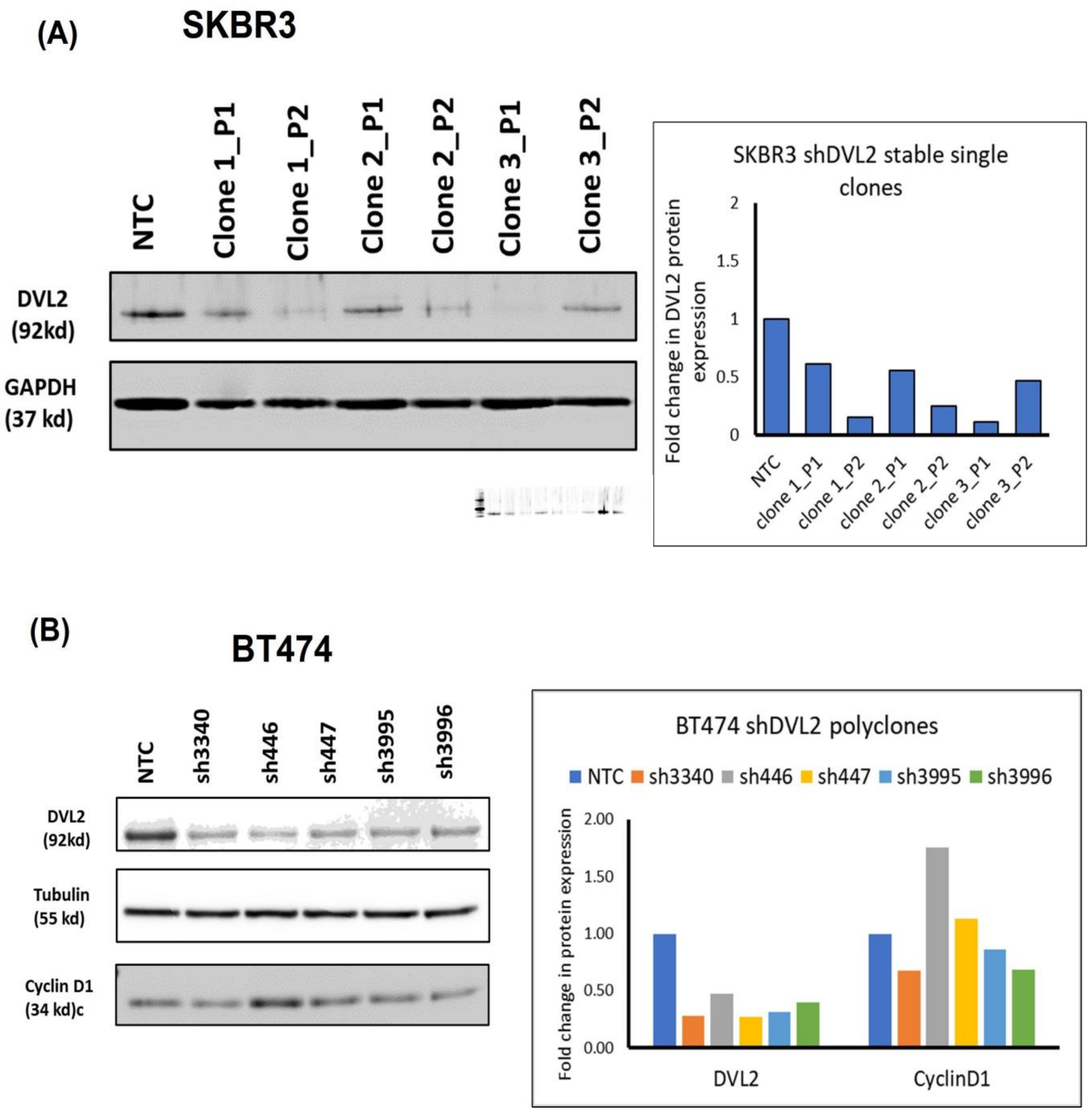

Fig S1b – Raw data for western blots in fig S1a

A. SKBR3

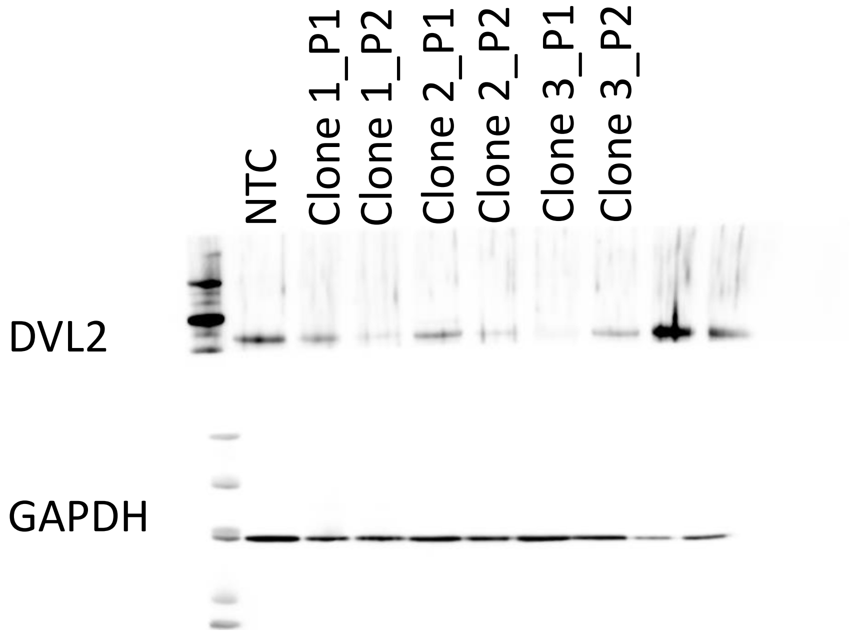

B. BT474

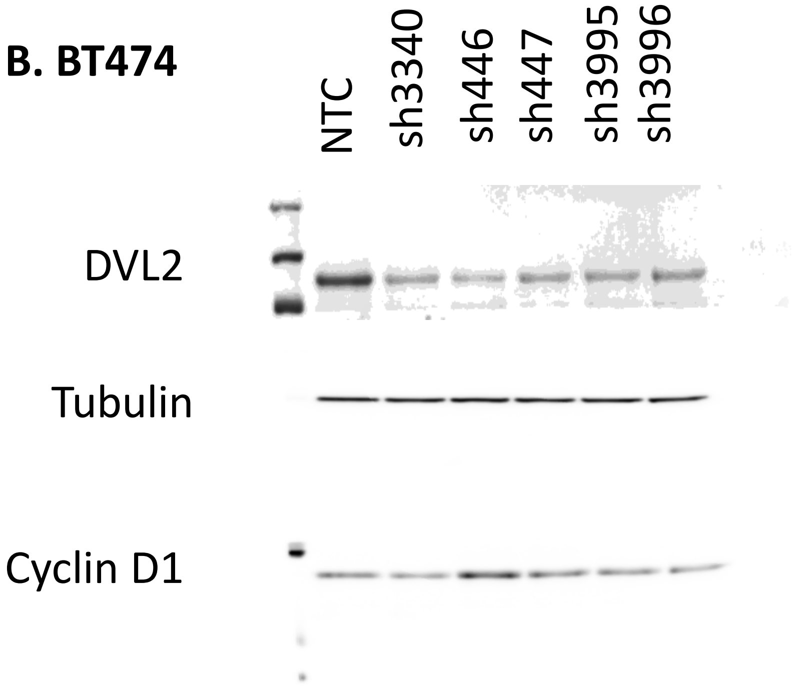

The molecular weight of each antibody is mentioned in the supplementary table S10

Figure S2a – Raw data for western blots in figure 2

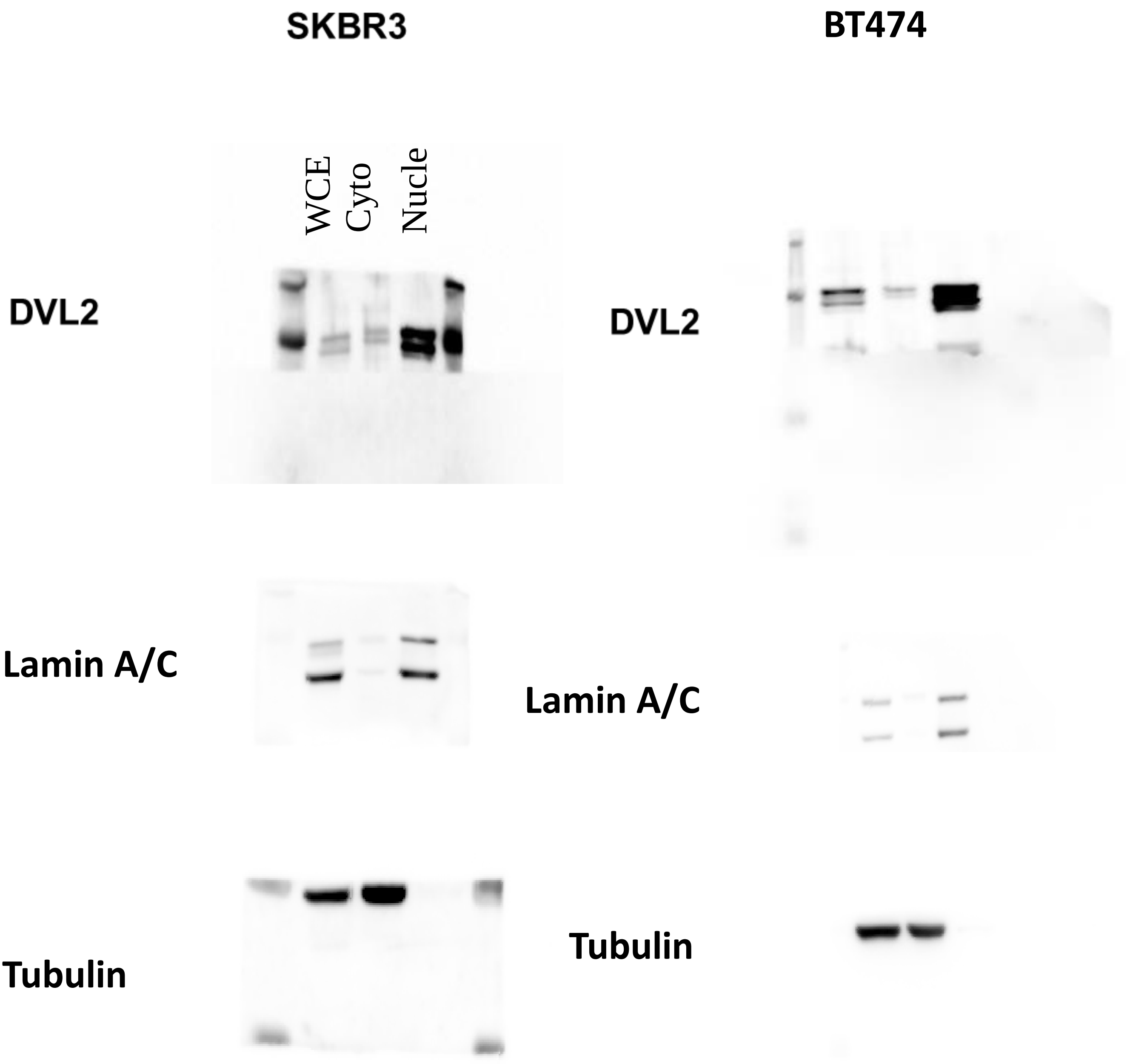

The molecular weight of each antibody is mentioned in the supplementary table S10. The blots were cut prior to hybridization with antibodies and cropped to improve the clarity and conciseness of the presentation.

Figure S2a – Raw data for ChIP data in figure 2

SKBR3

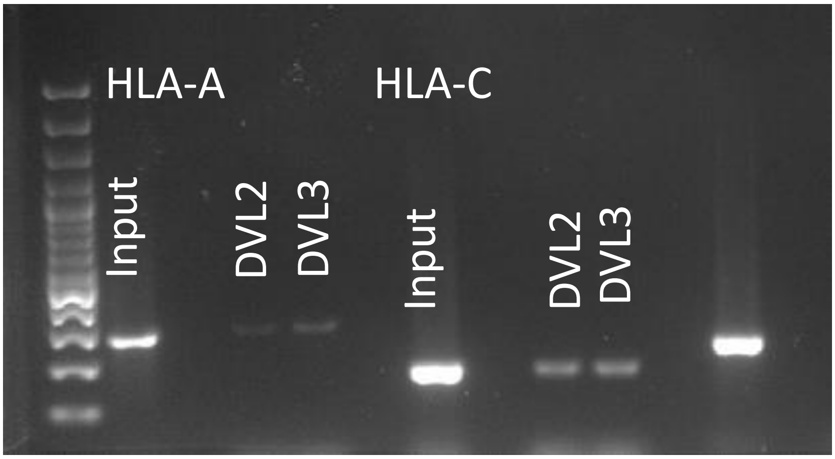

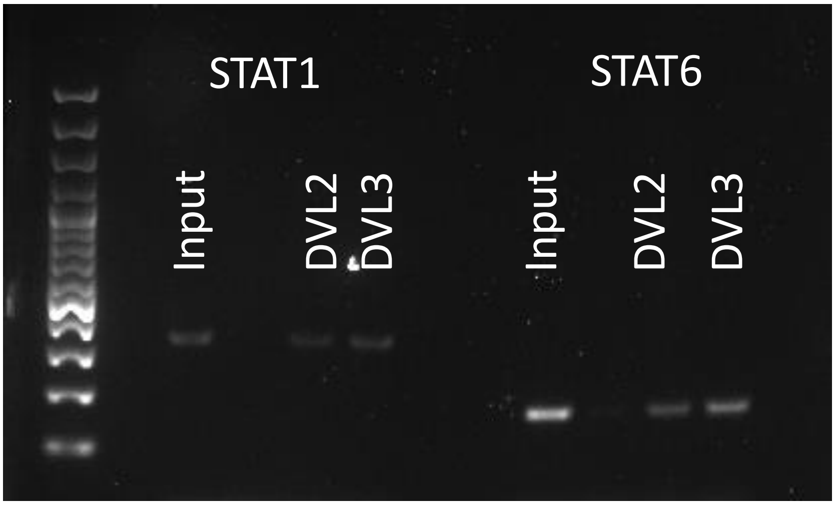

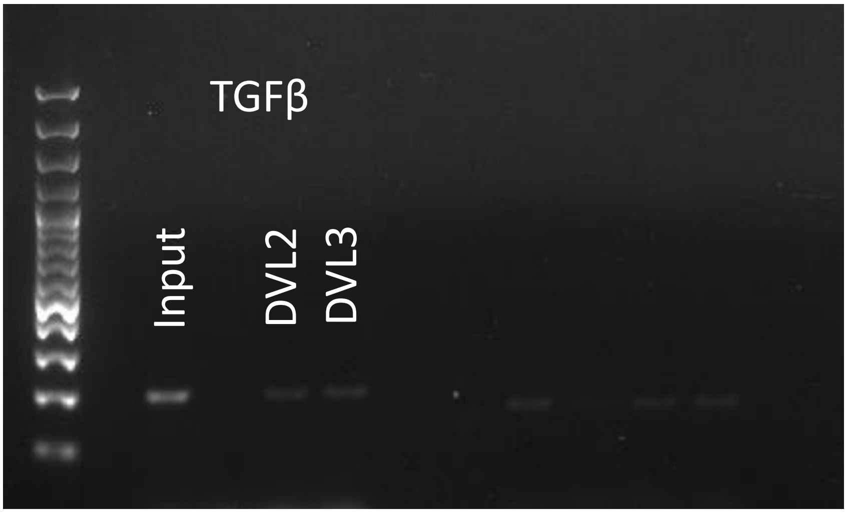

Figure S2b – Raw data for ChIP data in figure 2

BT474

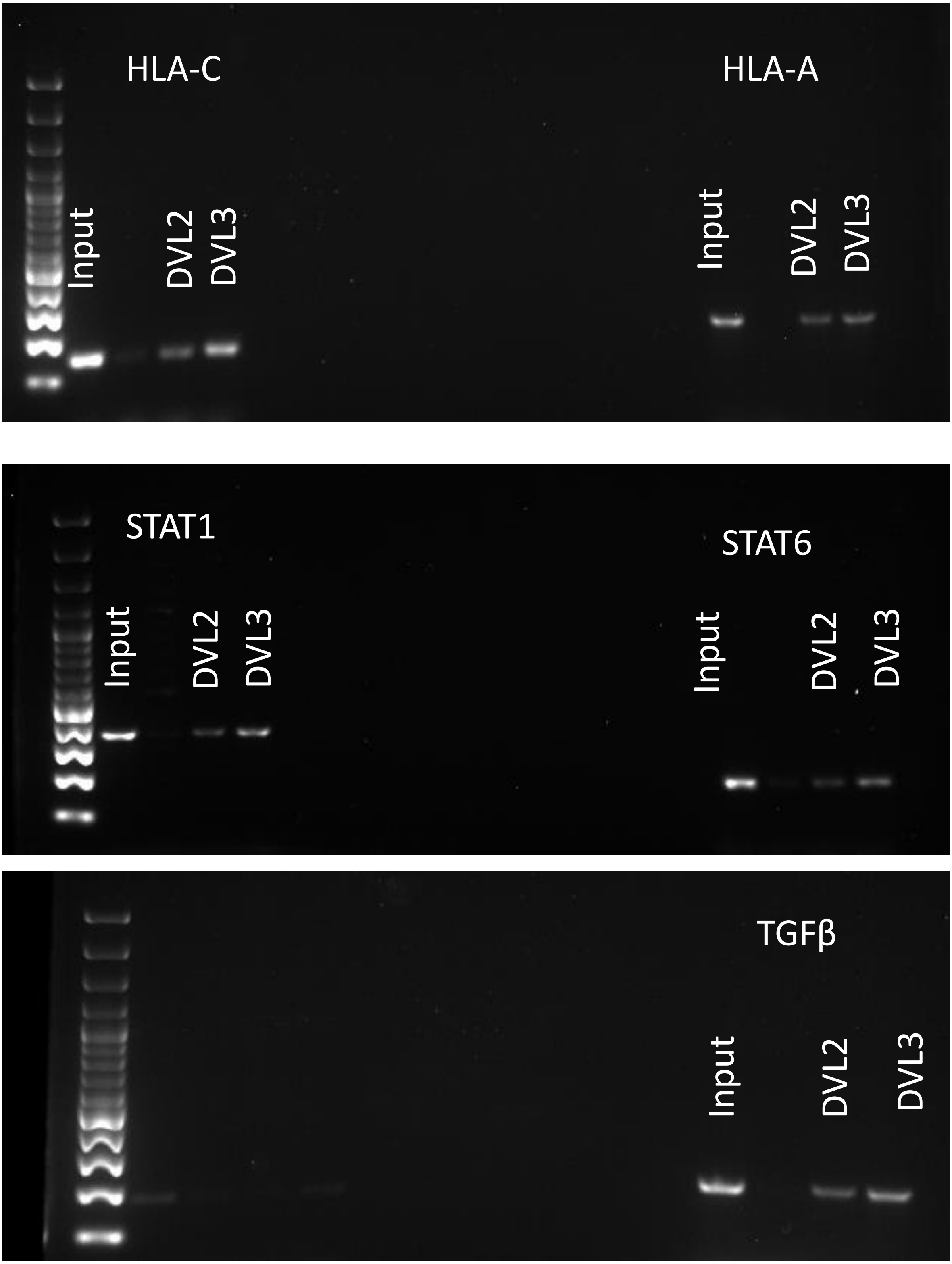

Figure S4a- Raw data for western blots in figure 4.

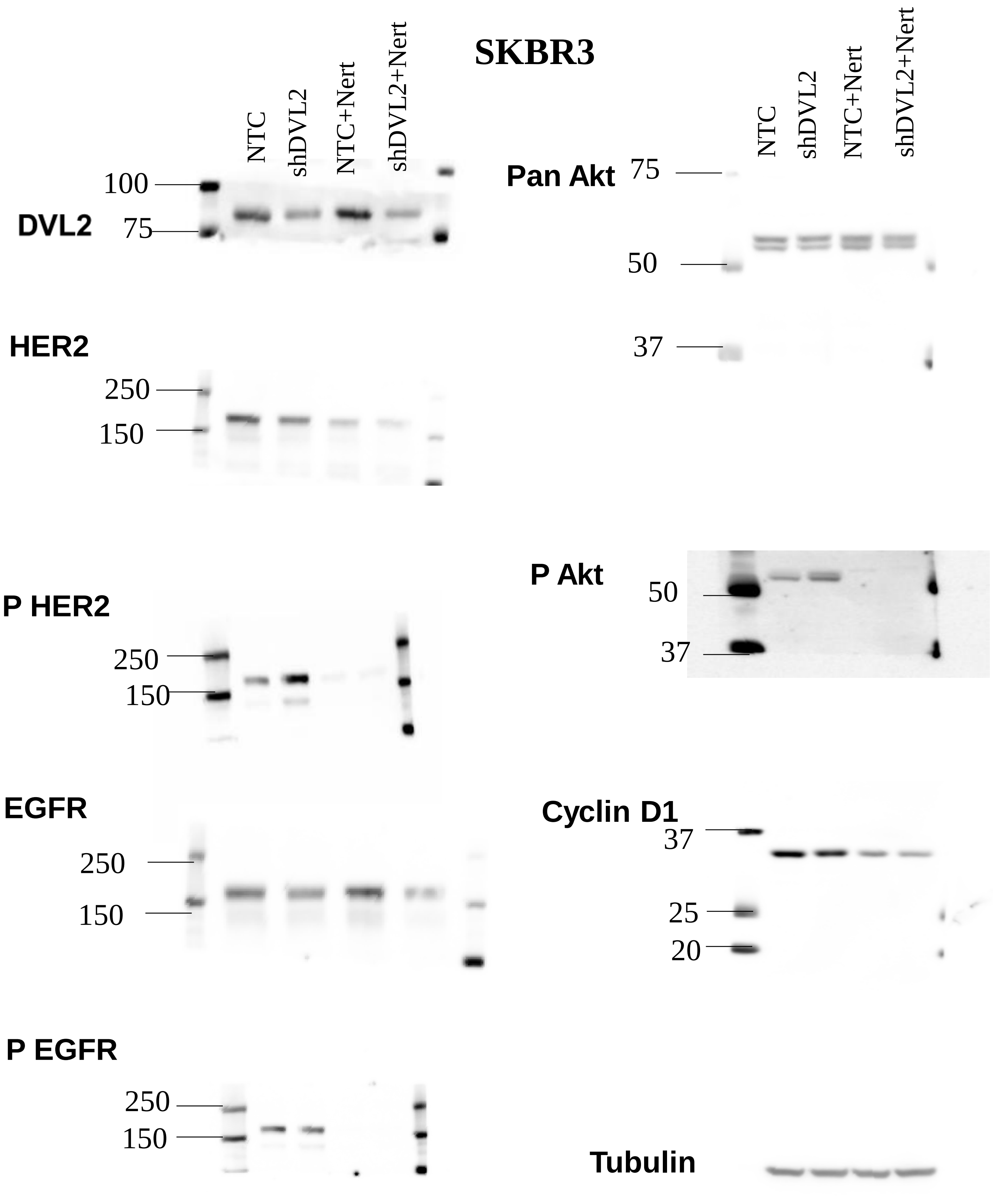

Figure S4b- Raw data for western blots in figure 4

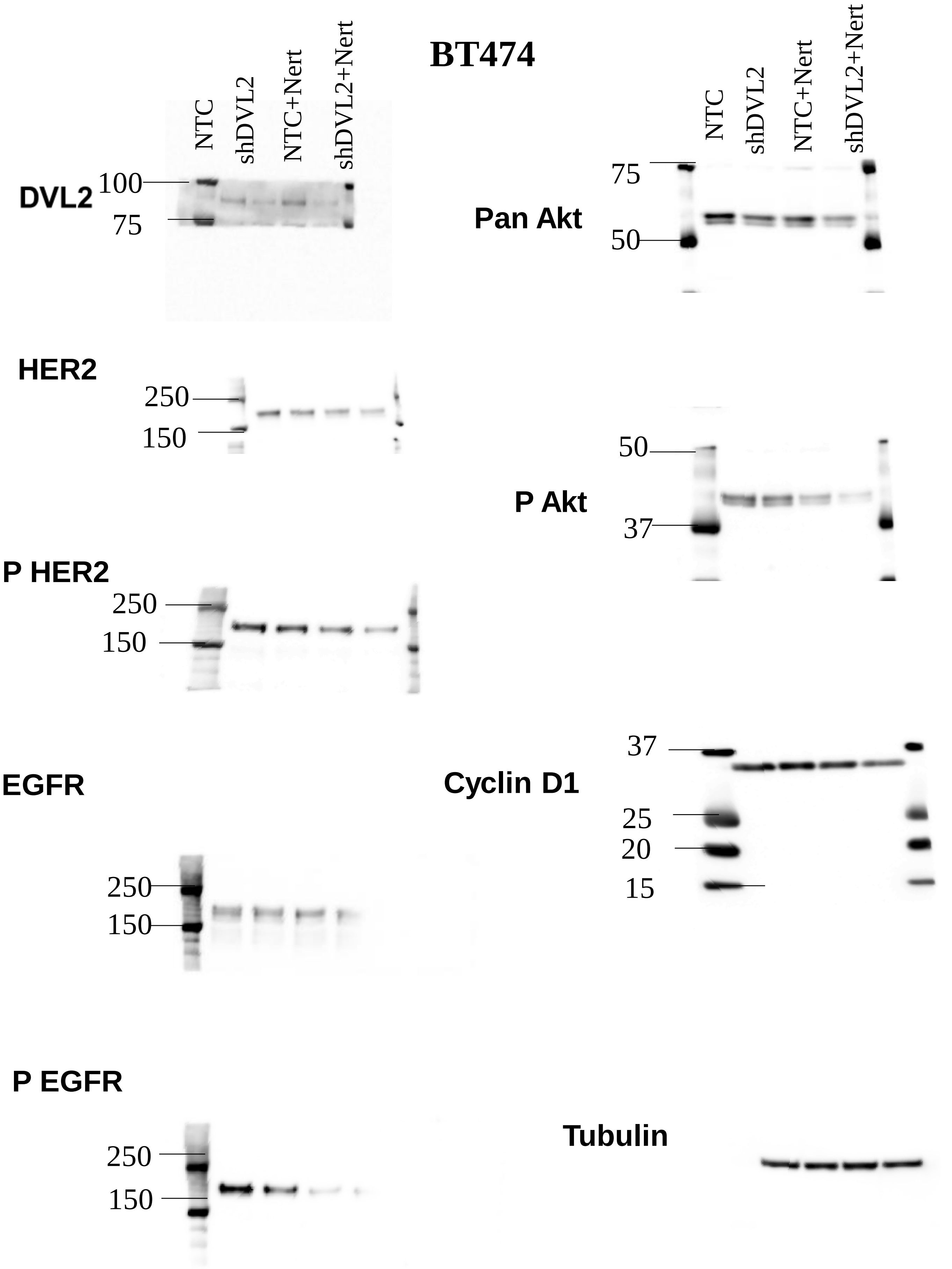

Raw data for westerns in fig S4a & S4b -

The molecular weight of each antibody is mentioned in the supplementary table S10. The blots were cut prior to hybridization with antibodies and cropped to improve the clarity and conciseness of the presentation.
