# Supplementary material for: Dishevelled 2 regulates cancer cell proliferation and T cell mediated immunity in HER2-positive breast cancer
Source: BMC Cancer. 2023 Feb 21;23:172. doi: 10.1186/s12885-023-10647-2 (PMC9942370; doi:10.1186/s12885-023-10647-2)
Supplement: Supplementary file 2 — Additional file 2: Table S1. Protein quantification of western blots of SKBR3 and BT474 cells expressing NTC vs shDVL2 treated with Neratinib. Table S2. Association between DVL-2 protein expression and clinicopathological parameters in HER2-positive breast cancer patient cohort at baseline (N = 24,*p < 0.05). Table S3. Association between DVL-2 protein expression and cancer survival predictors in HER2-positive breast cancer patient cohort (N = 24, p < 0.05). Table S4. Spearman’s rank correlation matrix for DVL2 and clinical parameters and/or survival predictors of breast cancer in 24 HER2-positive breast cancer patients after biopsy at baseline. Table S5. Spearman’s rank correlation matrix for DVL2 and clinical parameters and/or survival predictors of breast cancer at baseline in 14 HER2-positive breast cancer patients who received neoadjuvant chemotherapy (NAC). Table S6. Spearman’s rank correlation matrix for DVL2 and clinical parameters and/or survival predictors of breast cancer after resection in 14 HER2-positive breast cancer patients who received neoadjuvant chemotherapy (NAC). Table S7. Spearman’s rank correlation matrix for DVL2 and clinical parameters and/or survival predictors of breast cancer at baseline biopsy in 10 HER2-positive breast cancer patients who didn’t receive neoadjuvant chemotherapy (NAC). Table S8. ChIP primers used for the study. Table S9. mRNA Primers used for the study. Table S10. Antibodies used for the study. [file 12885_2023_10647_MOESM2_ESM.pdf]

Table S1 - Protein quantification of western blots of SKBR3 and BT474 cells expressing NTC vs shDVL2 treated with Neratinib

Protein expression analysis : SKBR3 - Fold change in expression with NTC as control							
	NTC	shDVL2	NTC+Nert	shDVL2+Nert			
DVL2	1	0.51	1.22	0.39			
HER2	1	0.7	0.27	0.14			
p-Her2	1	2.72	0.11	0.17			
EGFR	1	0.73	1.24	0.37			
p-EGFR	1	1.04	0	0			
pan-Akt	1	1	1.28	0.87			
p-Akt	1	2.49	0	0			
CyclinD1	1	0.88	0.37	0.26			
Protein expresssion analysis : BT474 - Fold change in expression with NTC as control							
	NTC	shDVL2	NTC+Nert	shDVL2+Nert			
DVL2	1	0.23	0.79	0.21			
HER2	1	0.99	1.1	0.63			
p-Her2	1	0.72	0.45	0.24			
EGFR	1	0.77	0.7	0.43			
p-EGFR	1	0.55	0.09	0.06			
pan-Akt	1	0.87	1.49	0.67			
p-Akt	1	0.55	0.44	0.18			
CyclinD1	1	0.99	0.71	0.54			

Table S2: Association between DVL-2 protein expression and clinicopathological parameters in HER2-positive breast cancer patient cohort at baseline (N=24,*p<0.05)

**Variables**	**DVL-2**		
	**N**	**Mean±SEM**	**p-value**
**Age**			
≤51	8	-0.1237 ± 0.1329	0.039*
>51	16	0.4136 ± 0.206	
**BMI**			
≤30	18	0.2709 ± 0.1454	0.769
>30	6	0.1253 ± 0.4527	
**ER status**			
+	15	0.2758 ± 0.2178	0.709
-	9	0.1656 ± 0.1931	
**PR status**			
+	9	0.241 ± 0.3633	0.978
-	15	0.2306 ± 0.1237	
**%Ki-67**			
<25	6	0.4152 ± 0.3778	0.578
≥25	17	0.1735 ± 0.1733	
**T stage**			
< T2	10	0.06733 ± 0.1779	0.331
≥ T2	14	0.3539 ± 0.2271	
**N stage**			
N0	12	-0.03621 ± 0.1757	0.074
≥ N1	12	0.5052 ± 0.2283	
**M stage**			
M0	20	0.2331 ± 0.1824	0.966
M1	4	0.2417 ± 0.07802	
**Neutrophil to lymphocyte Ratio (NLR)**			
<2	8	0.1055 ± 0.2677	0.567
≥2	15	0.3015 ± 0.2012	
**Platelet to lymphocyte Ratio (NLR)**			
<133	7	0.3697 ± 0.3185	0.606

≥133	16	0.1736 ± 0.1856	
**Neoadjuvant chemotherapy (NAC)**			
Yes	14	0.1683 ± 0.2183	0.604
No	10	0.3272 ± 0.208	

Table S3: Association between DVL-2 protein expression and cancer survival predictors in HER2-positive breast cancer patient cohort (N=24, p<0.05)

**Variables**	**DVL-2**		
	**N**	**Mean diff. ± SEM**	**p-value**
**Pathologic complete response (pcR)**			
Yes	7	-1.201 ± 0.151	0.885
No	5	-1.127 ± 0.460	
**Tumor infiltrating lymphocyte (TIL) score**			
Biopsy	14	-12.59 ± 3.927	0.004*
Resection	10	-8.744 ± 4.133	0.063
**% CD8α levels**			
Biopsy	14	-0.9869 ± 0.183	<0.0001*
Resection	10	0.214 ± 0.159	0.205

Table S4: Spearman's rank correlation matrix for DVL2 and clinical parameters and/or survival predictors of breast cancer in 24 HER2-positive breast cancer patients after biopsy at baseline

	**TIL score_baseline**			**DVL2_baseline**			**CD8a_baseline**		
	rho	rho.p	N	rho	rho.p	N	rho	rho.p	N
Age	-0.48	0.025	22	0.35	0.094	24	-0.30	0.156	24
Age >51 years	-0.39	0.071	22	0.37	0.075	24	-0.20	0.338	24
BMI	-0.17	0.447	22	-0.10	0.626	24	0.24	0.265	24
BMI >30	-0.26	0.249	22	-0.10	0.651	24	0.38	0.071	24
Clinical Stage	-0.13	0.563	22	0.18	0.400	24	-0.25	0.244	24
ER status	0.40	0.064	22	0.08	0.707	24	0.19	0.383	24
%Ki67 >25	0.37	0.094	21	-0.10	0.635	23	-0.31	0.145	23
M stage	0.00	1.000	22	0.10	0.652	24	-0.06	0.764	24
After resection_CD8a levels	0.13	0.725	10	-0.11	0.744	12	-0.19	0.561	12
Baseline_CD8a levels	-0.20	0.379	22	-0.27	0.206	24	1.00	na	na
After resection_DVL2 levels	0.31	0.387	10	-0.10	0.749	12	0.07	0.835	12
Baseline_DVL2 levels	-0.02	0.916	22	1.00	na	na	-0.27	0.206	24
After resection_TIL score	0.40	0.250	10	0.58	0.082	10	-0.56	0.097	10
Baseline_TIL score	1.00	na	na	-0.02	0.916	22	-0.20	0.379	22
N stage	0.22	0.334	22	0.40	0.055	24	-0.42	0.040	24
NAC_yes	0.19	0.386	22	-0.10	0.650	24	0.06	0.777	24
Neutrophil to lymphocyte Ratio (NLR)	-0.01	0.958	21	0.35	0.101	23	-0.32	0.143	23
pCR_cat_yes1_no0	-0.12	0.792	11	-0.30	0.354	13	0.13	0.724	13
Platelet to Lymphocyte Ratio (PLR)	-0.33	0.149	21	-0.08	0.723	23	0.20	0.353	23
PR status	-0.19	0.387	22	-0.17	0.433	24	0.47	0.022	24
Residual Tumor Burden Index	0.24	0.280	22	0.10	0.637	24	-0.01	0.955	24
T stage	-0.19	0.428	20	0.12	0.605	21	-0.05	0.829	21

Table S5: Spearman’s rank correlation matrix for DVL2 and clinical parameters and/or survival predictors of breast cancer at baseline in 14 HER2-positive breast cancer patients who received neoadjuvant chemotherapy (NAC)

	**TIL score_at biopsy**			**DVL2_at biopsy**			**CD8a_at biopsy**		
	rho	rho.p	N	rho	rho.p	N	rho	rho.p	N
Age	-0.50	0.100	12	0.50	0.069	14	-0.13	0.651	14
Age >51 years	-0.41	0.195	12	0.48	0.097	14	-0.05	0.871	14
BMI	-0.14	0.671	12	-0.06	0.829	14	0.48	0.081	14
BMI >30	-0.08	0.832	12	0.00	1.055	14	0.51	0.072	14
Clinical Stage	-0.42	0.195	12	0.19	0.555	14	-0.19	0.538	14
ER status	0.59	0.059	12	-0.19	0.555	14	0.17	0.571	14
%Ki67 >25	0.50	0.182	11	0.46	0.154	13	-0.46	0.154	13
After resection_CD8a levels	0.13	0.725	10	-0.11	0.744	12	-0.19	0.561	12
Baseline_CD8a levels	-0.05	0.874	12	-0.67	0.010	14	1.00	na	na
After resection_DVL2 levels	0.31	0.387	10	-0.10	0.749	12	0.07	0.835	12
Baseline_DVL2 levels	0.07	0.829	12	1.00	na	na	-0.67	0.010	14
After resection_TIL score	0.40	0.250	10	0.58	0.082	10	-0.56	0.097	10
Baseline_TIL score	1.00	na	na	0.07	0.829	12	-0.05	0.874	12
N stage	0.27	0.418	12	0.43	0.142	14	-0.43	0.134	14
Neutrophil to lymphocyte Ratio (NLR)	-0.06	0.847	12	0.58	0.033	14	-0.59	0.030	14
pCR_yes	-0.12	0.792	11	-0.30	0.354	13	0.13	0.724	13
Platelet to Lymphocyte Ratio (PLR)	-0.25	0.433	12	-0.24	0.409	14	-0.01	0.985	14
PR status	-0.17	0.619	12	-0.55	0.053	14	0.66	0.017	14
Residual Tumor Burden Index	0.29	0.400	11	0.17	0.622	13	0.00	1.025	13
T stage	-0.56	0.067	12	0.13	0.699	14	0.06	0.869	14

Table S6: Spearman’s rank correlation matrix for DVL2 and clinical parameters and/or survival predictors of breast cancer after resection in 14 HER2-positive breast cancer patients who received neoadjuvant chemotherapy (NAC)

	TIL score_after resection			DVL2_after resection			CD8a_after resection		
	rho	rho.p	N	rho	rho.p	N	rho	rho.p	N
Age	-0.17	0.638	10	-0.16	0.614	12	-0.34	0.275	12
Age >51 years	0.03	0.952	10	-0.19	0.589	12	-0.05	0.900	12
BMI	-0.30	0.387	10	-0.19	0.561	12	-0.31	0.331	12
BMI >30	0.00	1.050	10	0.05	0.933	12	-0.21	0.541	12
Clinical Stage	0.00	1.067	10	-0.26	0.485	12	0.55	0.076	12
ER status	0.04	0.933	10	0.00	1.091	12	0.32	0.348	12
%Ki67 >25	0.62	0.082	9	0.39	0.239	11	0.20	0.549	11
After resection_CD8a levels	0.32	0.368	10	-0.02	0.961	12	1.00	na	na
Baseline_CD8a levels	-0.56	0.097	10	0.07	0.835	12	-0.19	0.561	12
After resection_DVL2 levels	0.53	0.115	10	1.00	na	na	-0.02	0.961	12
Baseline_DVL2 levels	0.58	0.082	10	-0.10	0.749	12	-0.11	0.744	12
After resection_TIL score	1.00	na	na	0.53	0.115	10	0.32	0.368	10
Baseline_TIL score	0.40	0.250	10	0.31	0.387	10	0.13	0.725	10
N stage	0.24	0.500	10	-0.27	0.432	12	0.59	0.052	12
Neutrophil to lymphocyte Ratio (NLR)	0.28	0.431	10	0.01	0.991	12	-0.08	0.804	12
pCR_yes	-0.07	0.889	10	-0.07	0.876	12	-0.07	0.841	12
Platelet to Lymphocyte Ratio (PLR)	-0.62	0.061	10	-0.32	0.308	12	0.05	0.891	12
PR status	-0.42	0.246	10	0.00	1.063	12	-0.31	0.329	12
Residual Tumor Burden Index	0.19	0.586	10	0.17	0.596	12	0.06	0.851	12
T stage	0.04	0.950	10	0.00	1.067	12	0.38	0.230	12

Table S7: Spearman’s rank correlation matrix for DVL2 and clinical parameters and/or survival predictors of breast cancer at baseline biopsy in 10 HER2-positive breast cancer patients who didn't receive neoadjuvant chemotherapy (NAC)

	TIL score_at biopsy			DVL2_at biopsy			CD8a_at biopsy		
	rho	rho.p	N	rho	rho.p	N	rho	rho.p	N
Age	-0.41	0.237	10	0.09	0.800	10	-0.19	0.591	10
Age >51 years	0.06	1.000	10	-0.06	1.000	10	-0.41	0.400	10
BMI	-0.60	0.073	10	0.22	0.537	10	0.15	0.682	10
BMI >30	-0.52	0.178	10	-0.17	0.711	10	0.26	0.533	10
Clinical Stage	0.12	0.741	10	0.31	0.376	10	-0.36	0.302	10
ER status	-0.14	0.762	10	0.43	0.257	10	0.14	0.762	10
%Ki67 >25	0.17	0.690	10	-0.45	0.222	10	-0.38	0.310	10
M stage	0.21	0.610	10	0.14	0.762	10	-0.14	0.762	10
Baseline_CD8a levels	-0.50	0.144	10	0.31	0.387	10	1.00	na	na
Baseline_DVL2 levels	-0.43	0.218	10	1.00	na	na	0.31	0.387	10
Baseline_TIL score	1.00	na	na	-0.43	0.218	10	-0.50	0.144	10
N stage	0.12	0.736	10	0.31	0.390	10	-0.56	0.100	10
Neutrophil to lymphocyte Ratio (NLR)	0.28	0.463	9	-0.15	0.708	9	0.05	0.912	9
Platelet to Lymphocyte Ratio (PLR)	-0.72	0.056	9	0.52	0.222	9	0.52	0.222	9
PR_status	-0.52	0.178	10	0.70	0.044	10	0.26	0.533	10
T stage	-0.03	1.000	10	0.45	0.222	10	-0.24	0.548	10

Table S8 - ChIP primers used for the study

Gene	Forward Primer	Reverse Primer
HLA-A	TCTCCCTCTCCCAACCTACG	CAGAGGCGGTTTCCCTCC
HLA-C	CGGCGGTGTCGAAATACCTC	TCATGACGCGTCCCCAATTC
STAT1	AAAGACTCCCCAGGTGCCAA	GACGAGTTTAACGGCCTGCT
STAT6	CGCAGCACCTATCTGAGCA	CTTTTCTGTTGCCCCATCCA
TGFB1	GTTAGTGCGATCCAGAGAGGC	AACTCCAGGCATCTGAGGGA

Table S9 -	mRNA Primers used for the study

Gene	Forward Primer	Reverse Primer
DVL2	GAGCCAGGGGACATGCTTTT	GCTCATTTCGGGGGAGAGTG
HLA-C	GGTTGTCCTAGCTGTCCTTGG	TGCTAACAGGAACGCAGACA
TGFB1	ATGTCACTTGCCTCCACCCATC	ACCTGCTCTGAGGCCTGAAAG
STAT1	CCTTCAGTCTTTTCCAGCAGC	CTCATTCACATCTCTCAACTTCAC
STAT6	TTGAACTCGCTGGACAGAGC	TGCTCTCAAGGGTGCTGATG
DVL3	CTTCATCCGCCATACCGTCA	CGGGTACTGGTACGGGAAAG
CTNNB1	CTTGATATTGGTGCCCAGGGA	AACCACTCCCACCCTACCAAC
CCND1	TCTTGTGCCACAGATGTGAAGTT	GAGGCAGTCTGGGTCACACTT
POU5F1	CTTGAATCCCGAATGGAAAGGG	GTGTATATCCCAGGGTGATCCTC
VEGFA	CCCACTGAGGAGTCCACCAT	TTTCTTGCGCTTTCGTTTTT
MMP7	GCCCAGATGTGGAGTGCCAGA	TGAGCTCCTCGCGCAAAGCC
HER2	AGCCGCGAGCACCCAAGT	TTGGTGGGCAGGTAGGTGAGTT
AKT1	CATCTGTCACCAGGGGCTTAG	TGTAGCCAATGAAGGTGCCAT
B2M	CACCCCCACTGAAAAAGATG	ATATTAAAAAGCAAGCAAGCAGAA
GAPDH	TCGGAGTCAACGGATTTGGT	TTCCCGTTCTCAGCCTTGAC

Table S10 - Antibodies used for the study

Antibody	Dilution	Mol weight	catalog	Vendor	Species
DVL2	1 .200	92 kd	C375617	Lsbio	Rabbit
Lamin	1 .2000	74/63 kd	477S	Cell signaling	Mouse
Tubulin	1 .4000	55 kd	T7816	Sigma	Mouise
DVL2	1 .1000	92 kd	3224S	Cell signaling	Rabbit
HER2	1 .1000	185 Kd	4290s	Cell signaling	Rabbit
phospho-HER2	1 .1000	185 kd	2244S	Cell signaling	Rabbit
EGFR	1 .1000	175 Kd	4267s	Cell signaling	Rabbit
Phospho-EGFR	1 .1000	175 kd	3777S	Cell signaling	Rabbit
Pan Akt	1 .1000	60 kd	4691S	Cell signaling	Rabbit
phospho-Akt	1 .1000	60 kd	4060s	Cell signaling	Rabbit
CyclinD1	1 .10000	34 kd	ab134175	Abcam	Rabbit
